# Dielectric Confinement and Exciton Fine Structure in Lead Halide Perovskite Nanoplatelets

**DOI:** 10.3390/nano11113054

**Published:** 2021-11-13

**Authors:** Amal Ghribi, Rim Ben Aich, Kaïs Boujdaria, Thierry Barisien, Laurent Legrand, Maria Chamarro, Christophe Testelin

**Affiliations:** 1 LR01ES15 Laboratoire de Physique des Matériaux: Structure et Propriétés, Faculté des Sciences de Bizerte, Université de Carthage, Bizerte 7021, Tunisia; ghribiamal79@gmail.com (A.G.); ben.aich.rima07@gmail.com (R.B.A.); 2 Institut des NanoSciences de Paris, CNRS UMR 7588, Sorbonne Université, F-75005 Paris, France; thierry.barisien@insp.jussieu.fr (T.B.); laurent.legrand@insp.jussieu.fr (L.L.); maria.chamarro@insp.jussieu.fr (M.C.); christophe.testelin@insp.jussieu.fr (C.T.)

**Keywords:** perovskites, nanoplatelets, electronic and dielectric confinements, exciton energy, exciton fine structure

## Abstract

Owing to their flexible chemical synthesis and the ability to shape nanostructures, lead halide perovskites have emerged as high potential materials for optoelectronic devices. Here, we investigate the excitonic band edge states and their energies levels in colloidal inorganic lead halide nanoplatelets, particularly the influence of dielectric effects, in a thin quasi-2D system. We use a model including band offset and dielectric confinements in the presence of Coulomb interaction. Short- and long-range contributions, modified by dielectric effects, are also derived, leading to a full modelization of the exciton fine structure, in cubic, tetragonal and orthorhombic phases. The fine splitting structure, including dark and bright excitonic states, is discussed and compared to recent experimental results, showing the importance of both confinement and dielectric contributions.

## 1. Introduction

Two-dimensional colloidal nanostructures, also named nanoplatelets (NPLs), have attracted a large interest over the last decade. The first NPLs, similar to quantum wells, showing the growth control along one direction were synthesized in PbS [1] and then from II–VI semiconductors such as CdSe [2]. Such NPLs exhibit strong exciton confinement and outstanding optical and electronic properties for applications, such as a high absorption [3], large exciton binding energies [4], narrow and fast emission lines [5], or large two-photon absorption [6]. Moreover, the flexible chemical synthesis offers the opportunity to have free-standing nanostructures, adapted to device integration, while controlling the NPL thickness at the atomic layer and suppressing inhomogeneous broadening. More recently, the metal halide perovskites (MHP) have emerged as very attractive materials, first as bulk or thin films for photovoltaic, but also to elaborate highly tunable and emissive colloidal nanocrystals (NCs) [7]. As for II–VI compounds, the chemical synthesis has been shown to be effective to control the NC shape, such as NPL [8,9,10]. Being more defect tolerant than the II–VI semiconductors [11], the MHP might consist in candidates with superior properties for the implementation of lasers sources or more generally for photonic and polaritonic applications [12]. Perovskite NPLs with thicknesses of a few monolayers and a very large area can be easily incorporated in planar electro-optical devices with the advantage of a facile combination with other high-value functional materials and, in particular, with organic semiconductors.

Nonetheless, the optical emission spectrum, and its polarization properties, will fully depend on the band-edge excitons and its fine structure splitting (FSS). In perovskite materials, a reverse band ordering is observed for the band-edge states compared to more conventional II–VI semiconductors such as CdSe. Indeed, lowest energy excitons are the bound states, pairing an upper valence band (VB) hole $|j_{h}=1/2,j_{z}^{h}=\pm 1/2\rangle$ and an electron from the lowest split-off conduction band (CB) $|j_{e}=1/2,j_{z}^{e}=\pm 1/2\rangle$ leading to four excitonic states. The FSS is the result of the interplay of crystal field and the electron–hole (e-h) exchange interaction (EI). The latter one splits the four exciton states in one optically inactive state (dark state) with total angular momentum j=0, and three optically active states (bright states) having j=1. The FSS will be enhanced by the confinement, particularly strong in NPLs with a thickness of the order of few atomic layers. Moreover, because NPLs are free standing nanostructures, the dielectric effects induced by the dielectric mismatch between the inside and outside materials are going to increase the confinement and therefore the FSS [13,14]. Theoretically, the effect of the environment and dielectric mismatch has been addressed in CdSe NPLs either by **k.p** method [15,16] or tight binding theory [17] and more recently in halide inorganic perovskite NPL [18]. Neglecting the e-h EI, these authors have shown that the self-interaction with image charges is important and increases the exciton binding energy.

In this work, we focus on the calculation of the excitonic FSS in NPLs made of inorganic cesium lead halide perovskite. We use the **k.p** approach including dielectric effects under the image charge formalism and a variational excitonic wave function. Including both spatial and dielectric confinements, we deduce the eigen-energies of the band-edge excitonic states, and discuss the self and binding energies. The e-h EI, including short-range (SR) and long-range (LR) contributions, is then considered to calculate the FSS. In the following, by including both spatial and dielectric confinements, we first deduce the eigen-energies of the band-edge excitonic states and discuss their self and binding energies. Then, in a second part, the e-h EI, including short-range (SR) and long-range (LR) contributions, is considered to calculate the FSS and discussed. The theoretical methods are presented independently in each part.

## 2. Electronic and Dielectric Confinement Effects on the Exciton Energy

CsPbX${}_{3}$$\left(X=Br,I,Cl\right)$ are direct band gap semiconductors: the upper VB built from cationic Pb s-like orbitals and their states have a total angular momentum $j_{v}=\frac{1}{2}$, and the lower CB are built from the Pb p-like orbitals and a strong spin-orbit coupling splits the electron states with the total angular momentum $j_{e}=\frac{1}{2}$ (lower bands) and $j_{e}=\frac{3}{2}$ (upper bands). As previously mentioned, the band-edge exciton is formed by pairing an upper VB hole $|j_{h}=1/2,j_{z}^{h}=\pm 1/2\rangle$ and an electron from the lowest split-off CB electron $|j_{e}=1/2,j_{z}^{e}=\pm 1/2\rangle$. The hole states are deduced from the VB electron states $|j_{v}=\frac{1}{2},j_{z}^{v}=\pm \frac{1}{2}\rangle$ via the time-reversal operator I: $|j_{h}=1/2,j_{z}^{h}=\pm 1/2\rangle =I|j_{v}=\frac{1}{2},j_{z}^{v}=\mp \frac{1}{2}\rangle$. As the authors of [18] have done, in a first step of our calculations, the e-h EI will be neglected and we will consider four excitonic states fully degenerate.

The NPL is considered as a quasi-2D structure with a thickness L${}_{z}$ very small compared to the lateral sizes L${}_{x}$ and L${}_{y}$. The perovskite materials fills the volume defined by $-\frac{L_{z}}{2}<z<\frac{L_{z}}{2}$ and we will assume an infinite confinement potential outside of this volume.

### 2.1. Theoretical Methods

In absence of Coulomb interaction, the e-h pair system can be described by the Hamiltonian:

(1) $$H_{0}=\sum_{\ell =e,h}\left[T_{\ell }+V_{\ell }^{conf}\left(r_{\ell }\right)\right]+E_{g}$$

with $T_{\ell }=\left[\frac{{\left(p_{\|}^{\ell }\right)}^{2}}{2m_{\|}^{\ell }}+\frac{{\left(p_{z}^{\ell }\right)}^{2}}{2m_{z}^{\ell }}\right]$, the kinetic contribution of carrier *ℓ* (ℓ=e,h). *e* and *h* denote the electron and the hole, $m_{\|}^{\ell }$ is the in-plane mass of carrier *ℓ*, $m_{z}^{\ell }$ is the out-of-plane mass (along the strong confinement direction), and $p_{\|}^{\ell }$ and $p_{z}^{\ell }$ are the in- and out-of-plane momentum operators. $V_{\ell }^{conf}$ is the confining potential fixed by the band offset between the NPL and the barrier formed by the surrounding materials. We take $V_{\ell }^{conf}=0$ inside the NPL and $V_{\ell }^{conf}=+\infty$ outside. $E_{g}$ is the bulk band-gap energy. $r_{\ell }$ is the position vector of carrier *ℓ*, with in-plane and out-of-plane components $\rho_{\ell }=\left(x_{\ell },y_{\ell }\right)$ and z${}_{\ell }$.

The *e*-*h* state is defined by the wave function

(2) $$\Psi_{eh}\left(r_{e},r_{h}\right)=\frac{\exp(ik_{e}.\rho_{e})}{\sqrt{L_{x}L_{y}}}\frac{\exp(ik_{h}{.\rho }_{h})}{\sqrt{L_{x}L_{y}}}\chi \left(z_{e}\right)\chi \left(z_{h}\right)$$

with $\chi \left(z_{\ell }\right)=\sqrt{\frac{2}{L_{z}}}\cos\left(\frac{\pi z_{\ell }}{L_{z}}\right)$. $\Psi_{eh}\left(r_{e},r_{h}\right)=0$ outside of the NPL. $k_{\ell }$ is the kinetic momentum of carrier *ℓ* ($k_{e}=k_{h}=0$ in the ground state). In the limit of infinite lateral sizes, the *e*-*h* ground state energy, $E_{eh}$, reduces to the gap plus the kinetic contribution $\left(T=T_{e}+T_{h}\right)$:

(3) $$E_{eh}=E_{g}+\langle \Psi_{eh}|T|\Psi_{eh}\rangle =E_{g}+\frac{\hbar^{2}\pi^{2}}{2m_{\|}^{e}L_{z}^{2}}+\frac{\hbar^{2}\pi^{2}}{2m_{\|}^{h}L_{z}^{2}}=E_{g}+\frac{\hbar^{2}\pi^{2}}{2\mu_{\|}L_{z}^{2}}$$

where $\frac{1}{\mu_{\|}}=\frac{1}{m_{\|}^{e}}+\frac{1}{m_{\|}^{h}}$, with $\mu_{\|}$ being the in-plane reduced exciton mass.

In presence of Coulomb interactions, several contributions have to be included. The first one, independent of any dielectric mismatch, is a direct e-h Coulomb interaction $V_{C}\left(r_{e},r_{h}\right)$ which creates a bound state. Then, because the NPLs inside and outside may have different dielectric constants, new contributions have to be considered: (i) a single-particle self-energy which can modify strongly the single-particle confinement potential, for electron and hole, and (ii) a modification of the e-h Coulomb interaction $V_{C}$. The self-energy correction results from the interaction of particles having like charges and its incorporation in calculations tends to increase the single particle energies hence the band gap. The Coulomb interaction strength increases and is responsible for a larger e-h binding that oppositely translates into a reduction of the optical gap.

All these contributions can be described by introducing image charges. This image charge method is well established and reproduces the electric field induced by polarization charges at the interface between media with different dielectric constants [13,19]. This e-h pair system can then be described by the following Hamiltonian:

(4) $$H=\sum_{\ell =e,h}\left[T_{\ell }+V_{\ell }^{conf}\left(r_{\ell }\right)+V_{\ell }^{self}\left(r_{\ell }\right)\right]+V_{C}\left(r_{e},r_{h}\right)+E_{g}$$

The single particle potential (first right term) includes a new contribution, namely, a self-energy potential, $V_{\ell }^{self}$, induced by the interaction of the carriers with their image charges:

(5) $$V_{\ell }^{self}\left(r_{\ell }\right)=\frac{e^{2}}{4\pi \epsilon_{0}\epsilon_{1}}\sum_{n=\pm 1,\pm 2,\ldots }\frac{\eta^{\left|n\right|}}{2\left|z_{\ell }-{\left(-1\right)}^{n}z_{\ell }+nL_{z}\right|}$$

in which $\eta =\left(\epsilon_{1}-\epsilon_{2}\right)/\left(\epsilon_{1}+\epsilon_{2}\right)$, $\epsilon_{1}$, and $\epsilon_{2}$ are the dielectric constants inside and outside the NPL, respectively. If one gathers separately the terms with the same n-parity, one can show that

(6) $$V_{\ell }^{self}\left(r_{\ell }\right)=\frac{e^{2}}{8\pi \epsilon_{0}\epsilon_{1}L_{z}}\ln\left(\frac{1}{1-\eta^{2}}\right)+\frac{e^{2}}{4\pi \epsilon_{0}\epsilon_{1}}\sum_{p=0}^{+\infty }\frac{\left(2p+1\right)L_{z}}{{\left[\left(2p+1\right)L_{z}\right]}^{2}-4z_{\ell }^{2}}\eta^{2p+1}$$

as expected, this term is zero for η=0 corresponding to the case $\epsilon_{1}=\epsilon_{2}$.

The *e*-*h* Coulomb interaction is written as

(7) $$V_{C}\left(r_{e},r_{h}\right)=-\frac{e^{2}}{4\pi \epsilon_{0}\epsilon_{1}}\sum_{n=-\infty }^{n=+\infty }\frac{\eta^{\left|n\right|}}{{\left[{\left(\rho_{e}-\rho_{h}\right)}^{2}+{\left[z_{e}-{\left(-1\right)}^{n}z_{h}+nL_{z}\right]}^{2}\right]}^{1/2}}$$

If η=0, one has the usual expression $V_{C}\left(r_{e},r_{h}\right)=-\frac{e^{2}}{4\pi \epsilon_{0}\epsilon_{1}}\frac{1}{\left|r_{e}-r_{h}\right|}$. For η≠0, one can write this summation with two terms, $U_{C}$ and $W_{C}$, gathering respectively the components with even or odd n integers. One then obtains

(8) $$V_{C}\left(r_{e},r_{h}\right)=U_{C}\left(r_{e},r_{h}\right)+W_{C}\left(r_{e},r_{h}\right)$$

with

(9) $$U_{C}\left(r_{e},r_{h}\right)=-\frac{1}{{\left(2\pi \right)}^{3}}\int dq\left(\frac{e^{2}}{\epsilon_{0}\epsilon_{1}}\frac{1}{q^{2}}\right)\frac{1-\eta^{4}}{1+\eta^{4}-2\eta^{2}\cos\left(2q_{z}L_{z}\right)}\exp iq.\left(r_{e}-r_{h}\right)$$

(10) $$\begin{array}{ccc} W_{C}\left(r_{e},r_{h}\right) & = & -\frac{1}{{\left(2\pi \right)}^{3}}\int dq\left(\frac{e^{2}}{\epsilon_{0}\epsilon_{1}}\frac{1}{q^{2}}\right)\frac{2\eta \left(1-\eta^{2}\right)\cos\left(q_{z}L_{z}\right)}{1+\eta^{4}-2\eta^{2}\cos\left(2q_{z}L_{z}\right)}\\ & & \times \exp\left[iq_{\|}.\left(\rho_{e}-\rho_{h}\right)+iq_{z}\left(z_{e}+z_{h}\right)\right] \end{array}$$

in which $q=\left(q_{\|},q_{z}\right)=\left(q_{x},q_{y},q_{z}\right)$ denotes the wave vector in the reciprocal space and $q^{2}=q_{x}^{2}+q_{y}^{2}+q_{z}^{2}$.

Within the limit of a 2D system, this image-charge approach is compatible with the one which considers a dielectrically screened Coulomb interaction, commonly used in ab initio theoretical developments. Indeed, at the limit of large exciton components, here one finds the same asymptotic behavior for the enhancement factor of Coulomb interaction (=$\epsilon_{1}/\epsilon_{2}$) as computed for two point charges in a thin dielectric slab surrounded by a medium with a much lower dielectric constant by Keldysh in his pioneering works [20,21,22]. In these papers, as well as in [23] where the extreme case of an atomically thin dielectric is treated, for thin films the connection to the notion of ‘image charges’ is clearly and elegantly highlighted.

The Hamiltonian H can be solved by using a variational calculation and considering the following trial function:

(11) $$\begin{array}{ccc} \Psi (r_{e},r_{h}) & = & \frac{1}{\sqrt{L_{x}L_{y}}}N\left(a,\zeta \right)\chi \left(z_{e}\right)\chi \left(z_{h}\right)\exp\left(iK_{\|}.R_{\|}\right)\\ & & \times \exp-\frac{\sqrt{{\left(x_{e}-x_{h}\right)}^{2}+{\left(y_{e}-y_{h}\right)}^{2}+\zeta^{2}{\left(z_{e}-z_{h}\right)}^{2}}}{a} \end{array}$$

It is composed of the product of the single particle wave functions and a modified hydrogenoid wave function. This approach is commonly used for quantum wells, wires, or dots [24,25]. N(a,ζ) is the normalization factor, and *a* and ζ are the variational parameters. $R_{\|}$ is the in plane coordinate of the *e*-*h* pair center-of-mass and $K_{\|}$ is its kinetic momentum ($K_{\|}=0$ in the ground state). By minimizing $E_{X}=\langle \Psi |H|\Psi \rangle$, one obtains the exciton energy and the wave function.

### 2.2. Results and Discussion on Exciton Energy

**Figure 1:**
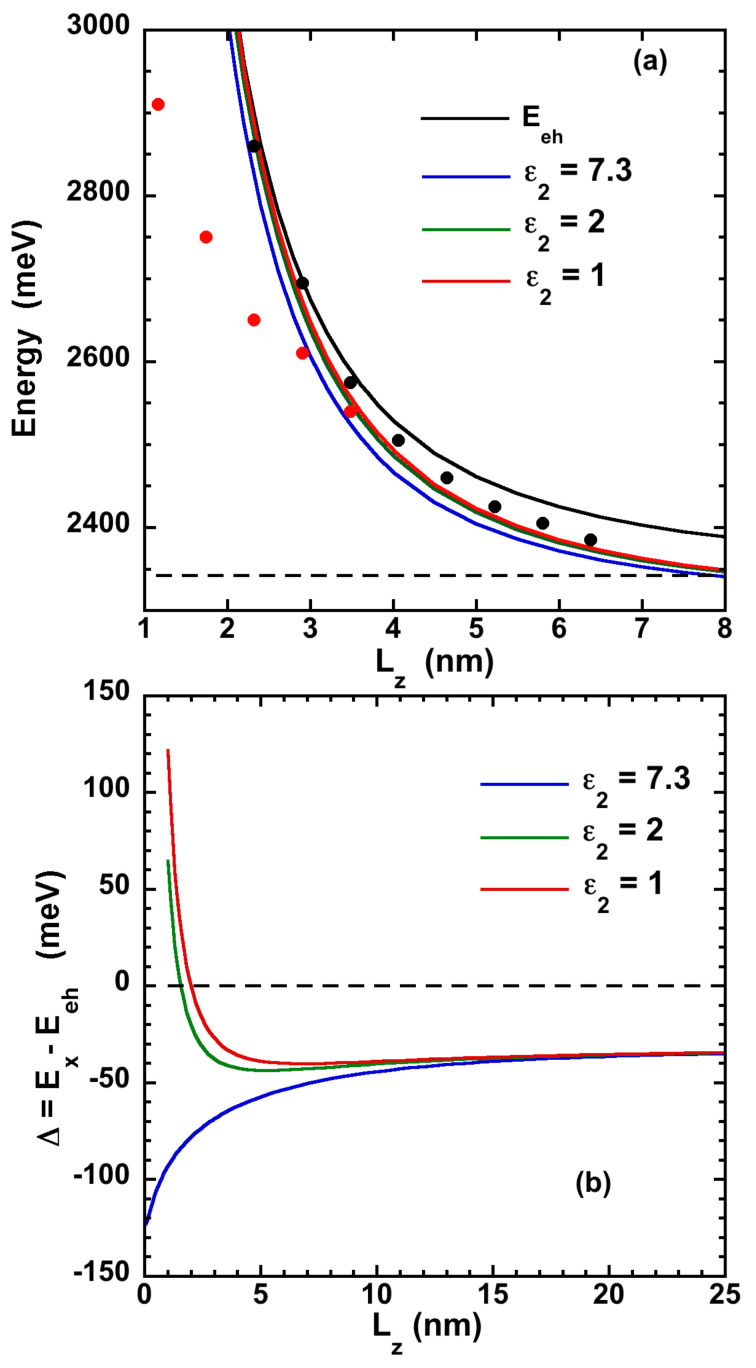
(**a**) Free e-h pair energy E${}_{eh}$ (black line) and excitonic energy E${}_{X}$ with different dielectric mismatches (the outside dielectric constant $\varepsilon_{2}$ varying from 7.3 to 1), for CsPbBr${}_{3}$. The dashed line correspond to the bulk gap energy E${}_{g}$. The black and red symbols are experimental data are from the works in [28,29], respectively. (**b**) Energy difference $\Delta =E_{X}-E_{eh}$ for different dielectric mismatches.

The energy can be compared to the energy $E_{eh}$, in absence of Coulomb interaction. These two energies are shown on Figure 1a, for CsPbBr${}_{3}$ (on Figure S2a for CsPbI${}_{3}$ and Figure S3a for CsPbCl${}_{3}$) and are also compared to experimental data.

We have used the parameters given in [26,27] and summarized them in Table S1. We have only reported exciton energies deduced from absorption measurements on CsPbBr${}_{3}$ NPL, because photoluminescence is generally Stokes shifted and underestimates the exciton energy. A very good agreement is obtained between the data from the work in [28] and the calculated exciton energy in presence of a dielectric mismatch, for 4 to 11 monolayers thicknesses. Nonetheless, the theory overestimates the emission measured in [29]. Such discrepancy may come from the NPL environment, such as stacking and finite barrier confinement [30]. Moreover, the assumption of an infinite barrier $\left(V_{\ell }^{conf}=+\infty \right)$ is no more valid for very small thicknesses. The difference $\Delta =\left(E_{X}-E_{eh}\right)$ is also shown in Figure 1b for CsPbBr${}_{3}$ (and in Figure S2b for CsPbI${}_{3}$ and Figure S3b for CsPbCl${}_{3}$). In absence of dielectric effect, namely, $\left(\epsilon_{1}=\epsilon_{2}\;\mathrm{and}\;\eta =0\right)$, Δ is the binding energy. It converges to 4R when $L_{z}\to 0$, with R=−32 meV, the bulk exciton binding energy (defined with a negative sign), as expected for purely 2D excitons in vacuum [31]. In presence of a dielectric mismatch, the thickness dependence of Δ is very different. We have considered two outside dielectric constants, $\epsilon_{2}=1$ (vacuum or air) and $\epsilon_{2}=2$ (close to the dielectric constant of glass, ligand or liquid). As observed on Figure 1b, Δ is almost constant when $L_{z}$ varies, down to $L_{z}\approx a_{X}$ ($a_{X}$ being the bulk exciton Bohr radius) and converges to *R* at large thickness.

In presence of the Coulomb interaction and a dielectric mismatch, $\Delta =\left(E_{X}-E_{eh}\right)$ includes three characteristic contributions. The first one is the modified e-h Coulomb interaction $\Delta_{C}=\langle \Psi |V_{C}\left(r_{e},r_{h}\right)|\Psi \rangle$.

**Figure 2:**
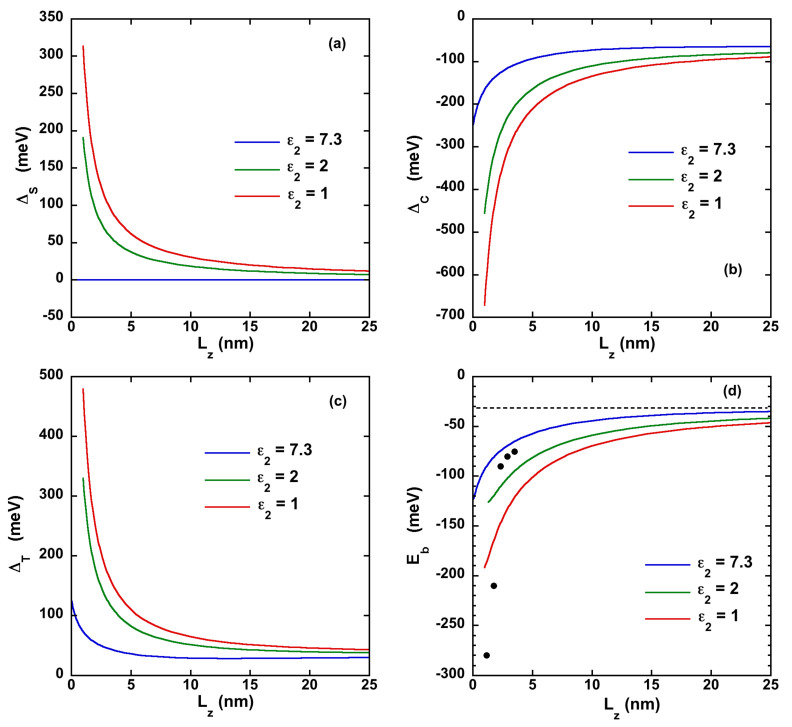
Different energy contributions induced by the Coulomb interaction and the dielectric effects in CsPbBr${}_{3}$ NPLs with different dielectric mismatches (the outside dielectric constant $\varepsilon_{2}$ varying from 7.3 to 1). (**a**) Summation of the electron and hole self energies $\Delta_{S}$. (**b**) Direct e-h Coulomb interaction $\Delta_{C}$. (**c**) Change in the kinetic energy $\Delta_{T}$ in presence of e-h coupling. (**d**) Binding energy $E_{b}$ of the e-h pair including the Coulomb interaction and the dielectric effects in CsPbBr${}_{3}$ NPLs with different dielectric mismatches. The black symbols are experimental values of the binding energy from in [29]. The dashed line corresponds to the bulk binding energy R = −32 meV.

The second one is the self energy $\Delta_{S}=\langle \Psi |\left(V_{e}^{self}+V_{h}^{self}\right)|\Psi \rangle$. The last one comes from the change in the kinetic energy: in presence of an e-h attractive coupling, the carrier wave function narrows leading to an increase of the kinetic term. This contribution can be defined as $\Delta_{T}=\langle \Psi |T|\Psi \rangle -\frac{\hbar^{2}\pi^{2}}{2\mu_{\|}L_{z}^{2}}$ ($\Delta_{T}=0$ in absence of e-h interaction). Δ is then equal to $\left(\Delta_{C}+\Delta_{S}+\Delta_{T}\right)$. The three contributions are displayed in Figure 2. For the sake of completeness, the self-energy potential functions (Equation (6)) and the enhancement factor associated with the modified Coulomb interaction (Equation (7)) are provided in the Supplementary Materials (Section S3) as a function of the dielectric contrast amplitude and NPL thickness.

The $\Delta_{\;C,\;S,\;T}$ quantities increase when L${}_{z}$ decreases and can be of the order of several hundred of meV, with $\Delta_{C}$ negative while $\Delta_{S}$ and $\Delta_{T}$ positive. They almost compensate each other down to $L_{z}\approx a_{X}$, explaining the stability of Δ in Figure 1b. This prediction is in agreement with the behavior experimentally observed on CsPbBr${}_{3}$ NPLs [28], with a thickness dependence for the excitonic energy such as $E_{X}=E_{g}+\Delta +\frac{\hbar^{2}\pi^{2}}{2\mu_{\|}L_{z}^{2}}$, with Δ quasi-constant.

The attractive e-h Coulomb interaction $\Delta_{C}$ is particularly important in presence of a dielectric mismatch. The strong increase of $\Delta_{C}$ with small L${}_{z}$ is due to (i) a strong enhancement of the carrier confinement by the self energy and (ii) an increase of the attractive e-h Coulomb interaction induced by the images charges (see Equation (7)).

The self-energy $\Delta_{S}$ is a single-particle contribution increasing the e (h) confinement energy and is not related to the e-h coupling. One will then define, in the presence of dielectric effects, the exciton binding energy $E_{b}=E_{X}-E_{eh}-\Delta_{S}=\Delta_{T}+\Delta_{C}$. The latter reduces to Δ in absence of dielectric effect or in the bulk situation (when $\Delta_{S}=0$). The dependence of the exciton binding energy is shown on Figure 2d with experimental values from Ref [29] showing the large increase of $E_{b}$ with dielectric effects (see Figure S4 for CsPbI${}_{3}$ and CsPbCl${}_{3}$). The ratio $\frac{E_{b}}{E_{eh}}$ increases as the NPL thickness decreases and is equal at ~4% for L${}_{z}$ = 2.32 nm (4 ML).

In the absence of the dielectric effect $\left(\epsilon_{1}=\epsilon_{2}\;\mathrm{and}\;\eta =0\right)$, $E_{b}=\Delta$ and one observes the usual behavior, from bulk to 2D; however, in the presence of a dielectric mismatch, a strong increase of E${}_{b}$ is visible, particularly at small thickness L${}_{z}$. This enhancement in the binding energy can be of particular importance in the temperature dependence of the exciton stability.

## 3. Electronic and Dielectric Confinement Effects on the Exciton Fine Structure

The e-h EI has not been considered in previous calculation. In moderately confined systems, this coupling is small compared to the confinement and direct e-h Coulomb interactions and can be neglected to estimate the excitonic energy. Nonetheless, we first show hereinafter that in few-layer confined systems, it might become comparable in strength to the exciton binding energy that results from the quantum and dielectric confinements. Second we address the issue of how the interplay of the e-h EI and crystal field leads to the exciton fine structure spectrum. The e-h EI modelization being indeed essential to describe the fine energetics and to position the dark singlet (optically inactive state) with respect to the bright (optically active) triplet. Experimental evidence of this exciton splitting and its enhancement with confinement has been shown for quantum wells [32] or colloidal NCs [33,34,35,36].

### 3.1. Theoretical Methods

The e-h EI was first calculated in bulk semiconductors by Pikus and Bir [37,38], and Denisov and Makarov [39]. The e-h EI was then considered in II-VI or III-V semiconductors, for low dimension systems, such as quantum wells [32,40] or quantum dots [14,41,42,43,44]. More recently, the e-h EI has been calculated in inorganic and hybrid halide perovskite NCs [45,46,47,48,49]. Two contributions have to be considered in the e-h EI: the so-called long-range (LR) and short-range (SR) parts (also named non-analytical and analytical). In most common semiconductors (such as II-VI and III-V compounds), a bright doublet and a dark doublet are present and the SR (LR) term is responsible for the dark-bright (bright-bright) splitting of the exciton states [44]. In bulk or NCs halide perovskite, the FSS is very different for two reasons: (i) one has three bright states and one single dark state, and (ii) both LR and SR interactions contribute to the dark–bright and bright–bright splittings, with comparable orders of magnitude [47,48].

Pairing CB electron $|j_{e}=1/2,j_{z}^{e}=\pm 1/2\rangle$ and VB hole $|j_{h}=1/2,j_{z}^{h}=\pm 1/2\rangle$, one can define the four e-h states $|j_{z}^{e}=\pm 1/2,j_{z}^{h}=\pm 1/2\rangle$. It is then possible to define the dark exciton, $|0_{D}\rangle$, and the three bright excitons $\left\{|+1\rangle ,|0_{B}\rangle ,|-1\rangle \right\}$:

(12) $$\left\{\begin{array}{c} |0_{D}\rangle =\frac{1}{\sqrt{2}}\left[|+\frac{1}{2},-\frac{1}{2}\rangle -|-\frac{1}{2},+\frac{1}{2}\rangle \right]\;\;\\ |0_{B}\rangle =\frac{1}{\sqrt{2}}\left[|+\frac{1}{2},-\frac{1}{2}\rangle +|-\frac{1}{2},+\frac{1}{2}\rangle \right]\;\;\\ |+1\rangle =|+\frac{1}{2},+\frac{1}{2}\rangle \;;\;|-1\rangle =|-\frac{1}{2},-\frac{1}{2}\rangle \end{array}\right.$$

The SR interaction is written as a contact interaction with the electron and hole spin Pauli operators [50]:

(13) $$H^{SR}=\frac{1}{2}C\left(I-\sigma_{e}.\sigma_{h}\right)\delta \left(r_{e}-r_{h}\right)$$

*C* is the SR exchange constant, derived from experimental data in [48] (*C* = 107.6 meV nm${}^{3}$) and is very close to the theoretical values (*C* = 92.2–105.7 meV nm${}^{3}$) obtained from DFT in [49], I is the 4×4 unit matrix, and $\sigma_{e}$ and $\sigma_{h}$ are the Pauli operators depicting the electron and hole spin.

In the basis $\left\{|+1\rangle ,|-1\rangle ,|0_{B}\rangle ;|0_{D}\rangle \right\}$, $H^{SR}$ is defined by the matrix

(14) $$H^{SR}=\frac{3}{2}\Delta_{SR}\pi a_{X}^{3}K\left[\begin{array}{cccc} \alpha^{2}+\beta^{2} & -\alpha^{2}+\beta^{2} & 0 & 0\\ -\alpha^{2}+\beta^{2} & \alpha^{2}+\beta^{2} & 0 & 0\\ 0 & 0 & 2\gamma^{2} & 0\\ 0 & 0 & 0 & 0 \end{array}\right]$$

with $\Delta_{SR}=\frac{2}{3}\frac{C}{\pi a_{X}^{3}}$ and $K=\int_{V}{\left|\Psi \left(r,r\right)\right|}^{2}dr$. From the $\Psi \left(r,r\right)$ expression, one deduces $K=\frac{3}{2}\frac{{\left|N(a,\zeta )\right|}^{2}}{L_{z}}$. $a_{X}$ is the bulk Bohr radius. The coefficients $\left(\alpha ,\beta ,\gamma \right)$ will depend on the crystal symmetry. For a cubic symmetry (with O${}_{h}$ as a point group), $\alpha^{2}=\beta^{2}=\gamma^{2}=\frac{1}{3}$. For a tetragonal crystal (D${}_{4h}$ point group), $\alpha^{2}=\beta^{2}=\frac{1}{2}\cos^{2}\theta$ and $\gamma^{2}=\sin^{2}\theta$. θ will be fixed by the spin orbit coupling, $\Delta_{C}$, and the tetragonal crystal field, *T*, following the relation $\tan2\theta =\frac{2\sqrt{2}\Delta_{C}}{\left(\Delta_{C}-3T\right)}$ [36,48] with 0<θ<π/2. In the orthorhombic symmetry (D${}_{2h}$ point group), the coefficients $\left(\alpha ,\beta ,\gamma \right)$ are defined in [51] and will depend on the orthorhombic crystal fields.

The expression of the LR interaction Hamiltonian has a symmetry similar to the SR interaction and is defined by a matrix:

(15) $$H^{LR}=\left[\begin{array}{cccc} \Sigma_{d} & \Sigma_{od} & 0 & 0\\ \Sigma_{od} & \Sigma_{d} & 0 & 0\\ 0 & 0 & \Sigma_{Z} & 0\\ 0 & 0 & 0 & 0 \end{array}\right]$$

The matrix coefficients can be derived from the general expression of the LR interaction [44,52]:

(16) $$H_{\begin{array}{c} m^{\prime }n^{\prime }\\ mn \end{array}}^{LR}\left(\begin{array}{cc} r_{e}^{\prime } & r_{h}^{\prime }\\ r_{e} & r_{h} \end{array}\right)=\sum_{i,j}Q_{\begin{array}{c} m^{\prime }In\\ In^{\prime }\;m \end{array}}^{ij}\frac{\partial^{2}}{\partial r_{e}^{i}\partial r_{e}^{j}}{\bar{V}}_{C}\left(r_{e}-r_{h}^{\prime }\right)\;\delta \left(r_{e}-r_{h}\right)\delta \left(r_{e}^{\prime }-r_{h}^{\prime }\right)$$

where ${\bar{V}}_{C}$ is derived from $V_{C}$ by changing $\epsilon_{1}$ in the denominator by $\epsilon_{X}$ which is the dielectric constant at the exciton resonance. Note that for the dielectric constant, we have considered the bulk values. Using DFT calculations, Sapori et al. [18] have shown, in CsPbI${}_{3}$, that for thicknesses larger or equal to 4 ML, the dielectric constant $\epsilon_{\infty }$ is very close to the bulk value in NPL. m, m’ (n, n’) label the Bloch states of the electron in the CB (the hole in the VB band), and $\left(r_{e},r_{e}^{\prime }\right)$ and $\left(r_{h},r_{h}^{\prime }\right)$ denotes the coordinates of the electrons and holes, respectively. Note that the time-reversal operator, I, leaves r unchanged but changes the kinetic momentum p and the angular momentum in their opposite.

$Q_{\begin{array}{c} m^{\prime }In\\ In^{\prime }\;m \end{array}}^{ij}$ is given by

(17) $$Q_{\begin{array}{c} m^{\prime }In\\ In^{\prime }\;m \end{array}}^{ij}=\frac{\hbar^{2}}{m_{0}^{2}}\frac{\langle m^{\prime }|p_{i}|In^{\prime }\rangle \langle In|p_{j}|m\rangle }{\left(E_{m}^{0}-E_{n}^{0}\right)\left(E_{m^{\prime }}^{0}-E_{n^{\prime }}^{0}\right)}\;,$$

where *m*${}_{0}$ denotes the free electron mass, $p_{i}$$\left(p_{j}\right)$ is the *i* (*j*) component of the p momentum, and $E_{\nu }^{0}$$\left(\nu =m,m^{\prime },n,n^{\prime }\right)$ is the $\nu^{th}$ band energy.

A full derivation of the LR Hamiltonian and the coefficients $\Sigma_{\lambda }$$\left(\lambda =o,od,z\right)$ is given in the Supplementary Information, taking into account the influence of the dielectric effects on ${\bar{V}}_{C}$. From the LR matrix, it is then possible to estimate the whole LR couplings:

(18) $$\left\{\begin{array}{l} \Sigma_{d}=\left(\alpha^{2}E_{P_{S,x}}I_{x}+\beta^{2}E_{P_{S,y}}I_{y}\right)\Lambda \frac{3\pi^{2}a_{X}^{3}}{L_{z}}{\left|N(a,\zeta )\right|}^{2}\;\;\;\;\\ \Sigma_{od}=\left(-\alpha^{2}E_{P_{S,x}}I_{x}+\beta^{2}E_{P_{S,y}}I_{y}\right)\Lambda \frac{3\pi^{2}a_{X}^{3}}{L_{z}}{\left|N(a,\zeta )\right|}^{2}\;\\ \Sigma_{Z}=2\gamma^{2}E_{P_{S,z}}I_{z}\Lambda \frac{3\pi^{2}a_{X}^{3}}{L_{z}}{\left|N(a,\zeta )\right|}^{2} \end{array}\right.$$

with $\Lambda =\frac{1}{3E_{g}^{2}}\frac{\hbar^{2}}{2m_{0}}\frac{e^{2}}{\epsilon_{0}\epsilon_{X}}\frac{1}{\pi a_{X}^{3}}$.

The integrals $I_{j}$$\left(j=x,y,z\right)$ and the Kane energies $E_{P_{S,j}}$ are given in the Supplementary Materials. The shape of the NPL is defined by the anisotropy ratio in planes x−y$\left(r=L_{y}/L_{x}\right)$ and x−z$\left(s=L_{z}/L_{x}\right)$. In the 2D limit, where $L_{z}<<L_{x}=L_{y}$, r=1, and s=0, one has then $I_{z}=\int du\frac{\sin^{2}u_{x}}{u_{x}^{2}}\frac{\sin^{2}u_{y}}{u_{y}^{2}}\frac{\sin^{2}u_{z}}{{\left(u_{z}^{2}-\frac{\pi^{2}}{4}\right)}^{2}}d\left(u_{z}\right)$ and $I_{x}=I_{y}=0$. Recalling that $d\left(u_{z}\right)=\frac{\left(1-\eta^{2}\right)}{1+\eta^{2}-2\eta \cos\left(2u_{z}\right)}$. In absence of dielectric mismatch $\left(\eta =0\right)$, $I_{z}=\frac{3}{2\pi }$. For a 2D-system, one has then $\Sigma_{d}=\Sigma_{od}=0$ and $\Sigma_{Z}=$ $2\gamma^{2}E_{P_{S,z}}I_{z}\Lambda \frac{3\pi^{2}a_{X}^{3}}{L_{z}}{\left|N(a,\zeta )\right|}^{2}$.

Finally, one can write the excitonic eigenvalues and eigenstates of $H=\left(H^{SR}+H^{LR}\right)$, noting that in cubic and tetragonal crystals, H is diagonal in the basis $\left\{|+1\rangle ,|-1\rangle ,|0_{B}\rangle ;|0_{D}\rangle \right\}$. 

In a cubic (O${}_{h}$) crystal, we have $\alpha^{2}=\beta^{2}=\gamma^{2}=1/3$ and the excitonic states are defined by

(19) $$\left\{\begin{array}{ll} \Sigma_{\pm }=\frac{3}{2}\Delta_{SR}\pi a_{X}^{3}\frac{{\left|N(a,\zeta )\right|}^{2}}{L_{z}} & \mathrm{for}\;|\pm 1\rangle \\ \Sigma_{Z}=\left[\frac{3}{2}\Delta_{SR}+2\pi E_{P_{S,z}}I_{z}\Lambda \right]\pi a_{X}^{3}\frac{{\left|N(a,\zeta )\right|}^{2}}{L_{z}} & \mathrm{for}\;|0_{B}\rangle \\ \Sigma_{0}=0 & \mathrm{for}\;|0_{D}\rangle \end{array}\right.$$

**Figure 3:**
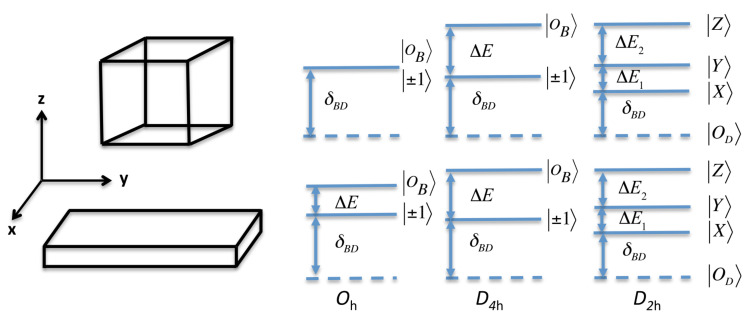
Energy labeling of the fine structure exciton states with cubic (O${}_{h}$), tetragonal (D${}_{4h}$), and orthorhombic (D${}_{2h}$) symmetry, for (**top**) a cube-shaped NC and (**bottom**) a quasi-2D NPL. In O${}_{h}$ symmetry, the three bright states are totally degenerate in a cube-shaped NC, while the degeneracy is partially lifted in the NPL. In tetragonal and orthorhombic symmetry, the FSSs have comparable ordering.

The degeneracy of the bright states is partially lifted by the LR contribution, as illustrated on Figure 3, and a bright doublet will appear in the emission with a bright–bright (BB) splitting $\Delta E=2E_{P_{S,z}}I_{z}\Lambda \pi^{2}a_{X}^{3}\frac{{\left|N(a,\zeta )\right|}^{2}}{L_{z}}$. This situation is characteristic of NPLs and must be compared to the situation of NCs with a confinement in three dimensions. For NCs with a cubic shape and a cubic crystal phase, the three bright states are degenerated. Only when the NC shape deviates from the perfect cubic shape, it is possible to observe the splitting of bright states into a doublet or a triplet states [45,51]. The bright–dark (BD) splitting is defined as $\delta_{BD}=\left(\Sigma_{\pm }-\Sigma_{0}\right)=\frac{3}{2}\Delta_{SR}\pi a_{X}^{3}\frac{{\left|N(a,\zeta )\right|}^{2}}{L_{z}}$ and it is only induced by the SR interaction. As we will see later on for other crystalline phases (D${}_{4h}$, D${}_{2h}$), LR does not contribute either to the $\delta_{BD}$ splitting of halide perovskite NPLs contrary to the case of bulk material or NCs. Furthermore, note that when $L_{z}$ decreases, the splittings increases not only because of the $L_{z}^{-1}$ dependence, but also because ${\left|N(a,\zeta )\right|}^{2}$ increases with confinement (see Figure S5).

### 3.2. Results and Discussion on Exciton FSS

**Figure 4:**
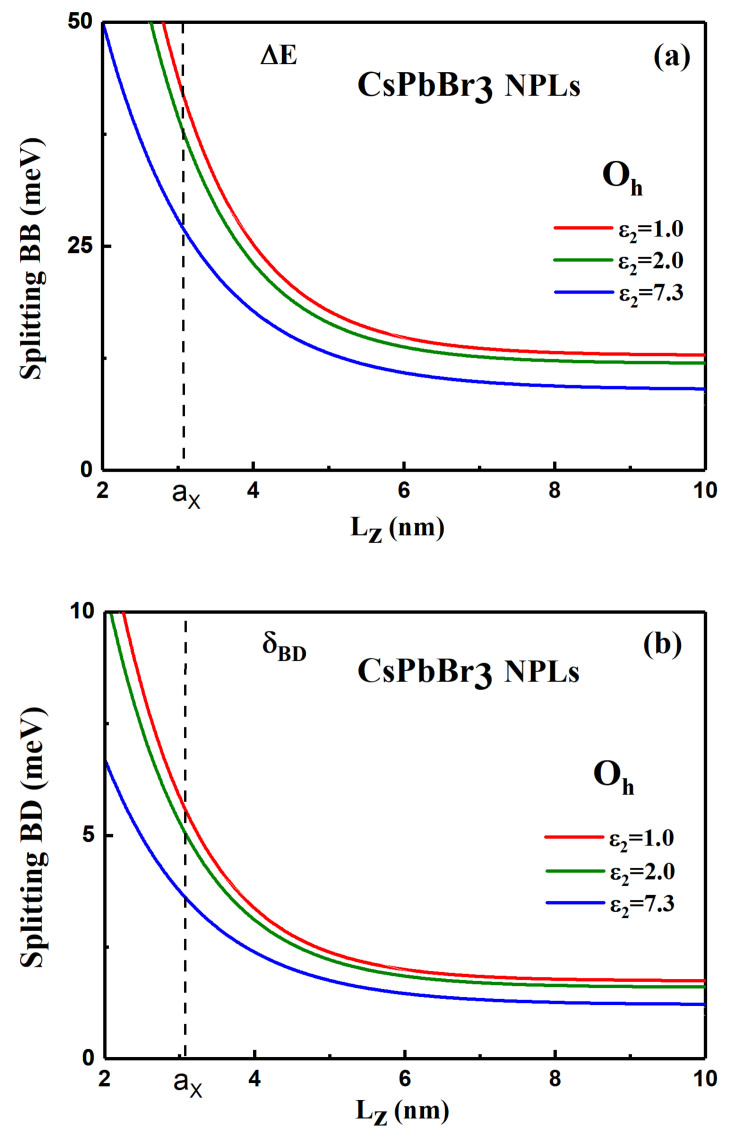
(**a**) Bright–bright splitting ΔE and (**b**) bright–dark splitting $\delta_{BD}$, in CsPbBr${}_{3}$ NPLs with a cubic symmetry and different dielectric mismatches (the outside dielectric constant $\varepsilon_{2}$ varying from 7.3 to 1). The vertical line indicates a size comparable to the exciton Bohr radius.

The behavior of the BB and BD splittings are shown in Figure 4 for CsPbBr${}_{3}$ NPLs, using the parameters given in Table S1 (see Figure S6 for CsPbI${}_{3}$ and Figure S7 for CsPbCl${}_{3}$ NPLs). Blue curves ($\epsilon_{2}=7.3$) correspond to an absence of dielectric mismatch. Vertical line gives the value of the exciton Bohr radius. Because the assumption of infinite potential of our model, the FSS for sizes smaller than the exciton Bohr radius can be overestimated, for small thicknesses. When the NPL thickness L${}_{z}$ decreases, one observes a strong enhancement of both splittings for L${}_{z}$ comparable or smaller than the bulk Bohr radius ($a_{X}$ = 3.07 nm). In presence of dielectric effects, the splitting are strongly enhanced by 50 to 70% for NPL thicknesses of the order of 4 to 6 monolayers (ML) and $\epsilon_{2}=1$ or 2 (for CsPbBr${}_{3}$, the atomic interplane distance is 0.58 nm; then 4 ML = 2.32 nm and 6ML = 3.48 nm) and even for a larger NPL thickness in the range $L_{z}$ = (3–4) $\times \;a_{X}$, the splitting is enhanced by ~30%. In the presence of a strong dielectric confinement, the BD splitting $\delta_{BD}$ is of the order of 10 meV for a thickness of $L_{z}$ = 4 ML (2.32 nm). Recently, Rossi and co-workers [53] claimed the observation of a dark state in time-resolved photoluminescence of CsPbBr${}_{3}$ NPLs with $\delta_{BD}$ = 11–22 meV for $L_{z}$ = 3–2 nm. These values are slightly larger or in the order of our calculations. The expected BB splitting, ΔE, is particularly important for small thicknesses less than 7 ML.

In a tetragonal (D${}_{4h}$) crystal, an elongation of the crystal lattice parameter appears along one direction, referred as Oz. The orientation of this axis, perpendicular or parallel to the NPL plane, will have an incidence on the energy order of the excitonic states and their polarization. High-Resolution Transmission Electronic Microscopy (HRTEM) imaging has recently been performed on large CsPbBr${}_{3}$ NPL, with a thickness of 2–3 nm [54], down to 7 ML [55] or 1 to 3 ML [56]. According to these studies, the *Z* axis will be perpendicular to the NPL larger plane, and this is the orientation fixed in the following analysis. Note that recent X-ray studies on CsPbBr${}_{3}$ NPLs have evidenced a different orientation of the tetragonal phase, with the *Z* axis along the NPL plane [57]. Such an orientation can be easily considered in our modelization and will be examined as a second step.

In the tetragonal phase, $\alpha^{2}=\beta^{2}=\frac{1}{2}\cos^{2}\theta$ and $\gamma^{2}=\sin^{2}\theta$, and the elongation direction of the crystal parallel to the confinement direction Oz $\left(I_{z}\neq 0\right)$ the excitonic states are defined by

(20) $$\left\{\begin{array}{ll} \Sigma_{\pm }=\frac{9}{4}\Delta_{SR}\pi a_{X}^{3}\frac{{\left|N(a,\zeta )\right|}^{2}}{L_{z}}\cos^{2}\theta & \mathrm{for}\;|\pm 1\rangle \\ \Sigma_{Z}=\left[\frac{9}{4}\Delta_{SR}+3E_{P_{S,z}}I_{z}\pi \Lambda \right]\pi a_{X}^{3}\frac{{\left|N(a,\zeta )\right|}^{2}}{L_{z}}2\sin^{2}\theta & \mathrm{for}\;|0_{B}\rangle \\ \Sigma_{0}=0 & \mathrm{for}\;|0_{D}\rangle \end{array}\right.$$

The tetragonal parameters, namely, the spin–orbit coupling $\Delta_{C}$, the tetragonal crystal field coupling *T*, the phase parameter θ, and the Kane energies $\left(E_{P_{S,\rho }},E_{P_{S,z}}\right)$, are given in Table S2 (see the Supplementary Materials).

As for cubic symmetry, the degeneracy of the bright states is partially lifted with an extra contribution from the SR interaction. The BB and BD splittings are written as

(21) $$\left\{\begin{array}{l} \Delta E=\left[\frac{9}{4}\Delta_{SR}\left(2\sin^{2}\theta -\cos^{2}\theta \right)+6E_{P_{S,z}}I_{z}\pi \Lambda \sin^{2}\theta \right]\pi a_{X}^{3}\frac{{\left|N(a,\zeta )\right|}^{2}}{L_{z}}\\ \delta_{BD}=\Sigma_{\pm }=\frac{9}{4}\Delta_{SR}\pi a_{X}^{3}\frac{{\left|N(a,\zeta )\right|}^{2}}{L_{z}}\cos^{2}\theta \;\;\;\;\;\;\;\;\;\;\;\;\;\;\;\;\;\;\;\;\;\;\;\;\;\;\;\;\;\;\;\;\;\; \end{array}\right.$$

**Figure 5:**
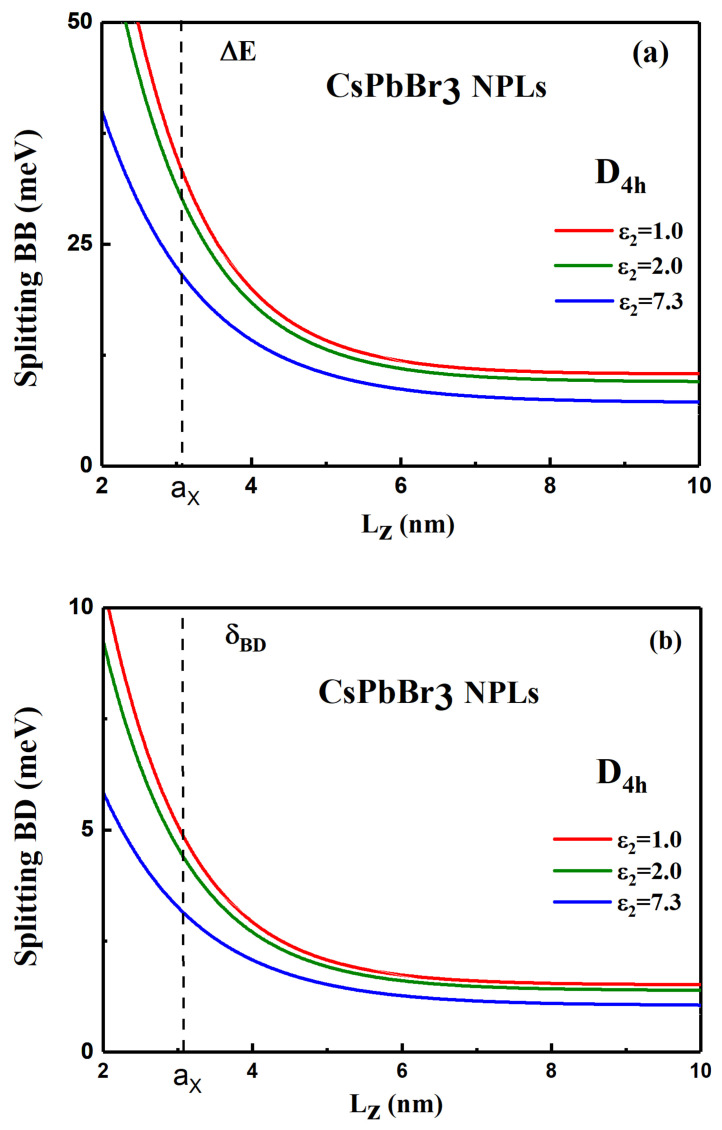
(**a**) Bright–bright splitting ΔE and (**b**) bright–dark splitting $\delta_{BD}$, in CsPbBr${}_{3}$ NPLs with a tetragonal symmetry and different dielectric mismatches (the outside dielectric constant $\varepsilon_{2}$ varying from 7.3 to 1). The vertical line indicates a size comparable to the exciton Bohr radius.

Both splittings are shown on Figure 5, versus the NPL thickness with and without dielectric effects (see Figure S6 for CsPbI${}_{3}$ and Figure S7 for CsPbCl${}_{3}$ NPLs).

Now, if the in-plane axes are *X* and *Z*, the exciton FSS will be derived from the previous equations, with $I_{x}=I_{z}=0$ and $I_{y}=\int du\frac{\sin^{2}u_{x}}{u_{x}^{2}}\frac{\sin^{2}u_{y}}{u_{y}^{2}}\frac{\sin^{2}u_{z}}{{\left(u_{z}^{2}-\frac{\pi^{2}}{4}\right)}^{2}}d\left(u_{z}\right)$.

In an orthorhombic (D${}_{2h}$) crystal, still assuming the *Z*-axis is perpendicular to the NPL, as in the tetragonal phase, the excitonic states are defined by

(22) $$\left\{\begin{array}{l} \Sigma_{X}=\frac{9}{2}\Delta_{SR}\pi a_{X}^{3}\frac{{\left|N(a,\zeta )\right|}^{2}}{L_{z}}\alpha^{2}\;\;\mathrm{for}\;|X\rangle =\frac{1}{\sqrt{2}}\left[|+1\rangle -|-1\rangle \right]\\ \Sigma_{Y}=\frac{9}{2}\Delta_{SR}\pi a_{X}^{3}\frac{{\left|N(a,\zeta )\right|}^{2}}{L_{z}}\beta^{2}\;\;\mathrm{for}\;|Y\rangle =\frac{1}{\sqrt{2}}\left[|+1\rangle +|-1\rangle \right]\\ \Sigma_{Z}=\left[\frac{9}{2}\Delta_{SR}+6E_{P_{S,z}}I_{z}\pi \Lambda \right]\pi a_{X}^{3}\frac{{\left|N(a,\zeta )\right|}^{2}}{L_{z}}\gamma^{2}\;\;\;\;\;\;\mathrm{for}\;|Z\rangle \\ \Sigma_{0}=0\;\;\;\;\;\;\;\;\;\;\;\;\;\;\;\;\;\;\;\;\;\;\;\;\;\;\;\;\mathrm{for}\;|0_{D}\rangle \;. \end{array}\right.$$

$\alpha^{2}$ and $\beta^{2}$ being different because of the orthorhombic crystal field, the FSS degeneracy is totally lifted as illustrated on Figure 3 ($\Delta E_{1}=\left(\Sigma_{Y}-\Sigma_{X}\right)$, $\Delta E_{2}=\left(\Sigma_{Z}-\Sigma_{Y}\right)$). With this convention and the Bloch states basis used to derived the EI interaction [46], the dipoles associated with *X*, *Y*, and *Z* oscillate along the *x*, *y*, and *z* physical axis, respectively. Using the realistic orthorhombic crystal field (80 meV) and the outside dielectric constant ($\epsilon_{2}=1$), we estimate the *X*-*Y* splitting to be $\Delta E_{1}$ = 1.9 meV for $L_{z}$ = 3 nm and $\Delta E_{1}$ = 1.4 meV for $L_{z}$ = 4 nm. In absence of dielectric mismatch ($\epsilon_{2}=\epsilon_{1}$ = 7.3), we get 1.4 meV as *X*-*Y* splitting for $L_{z}$ = 3 nm and 0.9 meV for $L_{z}$ = 4 nm.

As we have already underlined, the $\delta_{BD}$ splitting contains only a SR contribution to the e-h EI. $\Delta_{SR}$ in bulk semiconductors increases with decreasing value of $a_{X}$ and halide perovskite materials show the same behavior [48]. LR contribution increases also with decreasing value of $a_{X}$. Finally, for a given NPL size and crystal structure the BB and BD splittings increase with reduced Bohr radius of the considered perovskite compound and then both splittings increase with the halide atom as follows Cl > Br > I (see Figures S6 and S7 in Supplementary Materials).

In this work, we obtain for NPLs with $\frac{L_{z}}{a_{X}}=0.65$ an increase of $\delta_{BD}$ that does not exceed ten times the values of $\delta_{BD}$ for CsPbBr${}_{3}$ bulk material (about 1 meV). These values can be compared to CdSe NPLs which have been intensively studied. In the last years, a $\delta_{BD}$ splitting of 5 meV, 40 times larger than the CdSe bulk (0.13 meV) has been reported in very narrow NPLs $\left(\frac{L_{z}}{a_{X}}=0.16\right)$ [58]. To our knowledge, there are no theoretical or experimental results concerning the BB splittings for CdSe NPLs, however calculations and experimental values for very small CdSe NCs are provided in [59] and the references therein. We underline that very large splittings are obtained in general and specially for small NCs. The BB splitting is about 70 to 80 meV (theoretical results) or between 55 and 85 meV (experimental results) when the NC size is equal to 1.5 nm. The bulk $\delta_{BD}$ splitting being much smaller in CdSe than in CsPbBr${}_{3}$, large $\delta_{BD}$ can then be expected in the latter compound.

According to the FSS determined in this work, one might basically expect that a single NPL response, measured in micro-photoluminescence, is composed of two or three emission lines with associated states shown in Figure 3. A summary of the possible energy level configurations with respect to the position of the c crystallographic axis (in D${}_{2h}$ and D${}_{4h}$ symmetry) is provided in the Supplementary Materials (see Section S9). However, the large inter bright-states splitting ΔE or $\Delta E_{2}$$\left(\Delta E\;\mathrm{and}\;\Delta E_{2}\gg \Delta E_{1}\right)$ as well as the specific geometry of the system might lead to significantly affected responses. Three effects will indeed combine: first the rapid thermalization will drain states populations towards the lowest state of the fine structure manifold (through ultrafast non radiative processes), second the local field effect in the strongly confined direction will additionally reduce the probability that the highest energy states emit light [60]. Finally, as photons are collected by an objective having a finite angle of acceptance, dipoles oriented along the optical axis will very poorly contribute to the detected emission.

For NPLs laying flat, the photoluminescence is thus expected to be dominated by (i) a single peak for a D${}_{4h}$ symmetry with Z (or c axis) orthogonal to the NPL plane (an isotropic distribution of the emission intensity is then expected through a linear analyzer as light is then emitted by a degenerate in-plane doublet) and (ii) a weakly non-degenerate doublet $\left(\Delta E_{1}\ll \Delta E_{2}\right)$ with components of crossed polarizations ($D_{4h}$ symmetry with Z in the NPL plane or $D_{2h}$ symmetry, whatever Z orientation). This latter case can be spectrally evidenced if the NPLs can be dispersed (and the “single object” configuration reached), and if $\Delta E_{1}$ is less than the homogeneous linewidth. Such a doublet might indeed have been observed in PL-microscopy experiments in CsPbBr${}_{3}$ NPLs [61] or 2D perovskite like (PEA)${}_{2}$PbI${}_{4}$ [62].

For NPLs lying on their edge, the same considerations apply: in all cases, a single linearly polarized line is expected in a micro-photoluminescence experiment.

Figure S8 provides an illustration of how the different effects combine to shape the exciton fine structure spectrum with numerical estimations of the expected attenuation for some of the lines (see also the Supplementary Materials, Section S10, for the calculation of the screening factors that come as corrections to the oscillator strengths).

In absorption experiments performed in NPL ensemble, the local fields generated in the NPL will decrease the coupling amplitude between light and the dipole oriented along the more confined dimension. As a consequence, the full exciton bright state spectrum will be difficult to observe in absorption too. A strong weakening of the high energy absorption transition associated to the dipole oriented along the more confined dimension (ground state to $|0_{B}\rangle$ or $|Z\rangle$ transitions) is indeed expected. Taking into account the inhomogeneous broadening we will finally obtain an asymmetry in the absorption profile consistent with the experimental observations [28].

## 4. Conclusions

In conclusion, we have developed a description of the excitonic states in inorganic lead halide NPLs, focusing on the influence of the dielectric effects, both on the energy levels and the FSS. The knowledge of these two exciton characteristics will benefit mainly two different fields of the perovskite material applications. Indeed, predicting the emission wavelength of a NPL as a function of its thickness and composition is necessary in the field of nanophotonics applications like light-emitting diodes (LED) or lasers devices. The knowledge of FSS is of prime importance otherwise to design quantum devices like optical sensors or single-photon sources. In the presence of Coulomb interactions and dielectric mismatches, the energy levels are sensitive to three important contributions (e-h Coulomb attraction, single-particle self-energy, and kinetic energy) which mostly compensate each other, leading to a quasi-constant red-shift Δ of the free e-h pair energy, recently observed in CsPbBr${}_{3}$ NPLs [28]. Due to the 2D geometry, the excitonic bright triplet degeneracy is partially lifted in the cubic phase, a behavior different from what has been observed in cubic-shaped nanocrystals where the three bright states $\left(|+1\rangle ,|-1\rangle ,|0_{B}\rangle \right)$ are fully degenerate. As for perfect cubic-shaped nanocrystals, the bright state’s degeneracy is totally lifted in the orthorhombic phase. A strong enhancement of the FSS is predicted in the presence of dielectric effects, favored by a stronger confinement of the exciton wave function and a modification of the LR e-h interaction as modeled in this work. Furthermore, this work shows the importance of the NPL environment on the exciton energy levels and the FSS; this should be taken into consideration in future analyses of experimental results.

## Data Availability

The data presented in this study are available on request from the corresponding author.

## Supplementary Materials

The following are available online at https://www.mdpi.com/article/10.3390/nano11113054/s1, Table S1: Summary of basic physical parameters used in calculating the excitonic fine structure splittings. Figure S1: (Upper panel) Enhancement factor as the ratio of the modified Coulomb potential to the usual e-h electrostatic potential (median NPL plane) in CsPbBr${}_{3}$; (Lower panel) Plots of the self-energy potential in a CsPbBr${}_{3}$ NPL as a function of the NPL thickness and outer dielectric constant. Table S2: Numerical values of the tetragonal parameters used in this work. They are determined from the 16-band k.p model. Figure S2: (a) Free e-h pair energy $E_{eh}$ and excitonic energy $E_{X}$ with different dielectric mismatches, for CsPbI${}_{3}$; (b) Energy difference $\Delta =E_{X}-E_{eh}$ with different dielectric mismatches. Figure S3: (a) Free e-h pair energy $E_{eh}$ and excitonic energy $E_{X}$ with different dielectric mismatches, for CsPbCl${}_{3}$; (b) Energy difference $\Delta =E_{X}-E_{eh}$ with different dielectric mismatches. Figure S4: Binding energy $E_{b}$ of the e-h pair including the Coulomb interaction and the dielectric effects with different dielectric mismatches, in (a) CsPbI${}_{3}$ NPLs and (b) CsPbCl${}_{3}$ NPLs. Figure S5: Normalization factor N(a,ζ) of the trial function versus the thickness $L_{z}$ with different dielectric mismatches, for CsPbBr${}_{3}$ NPL; (b) Effective Bohr radius normalized by the bulk Bohr radius $a_{X}$. Figure S6: Bright-Bright splitting ΔE and Bright-Dark splitting $\delta_{BD}$, in CsPbI${}_{3}$ NPLs with an $O_{h}$ cubic symmetry and a $D_{4h}$ tetragonal symmetry. Different dielectric mismatches are considered, the outside dielectric constant $\epsilon_{2}$ varying from 10 to 1. Figure S7: Bright-Bright splitting ΔE and Bright-Dark splitting $\delta_{BD}$, in CsPbCl${}_{3}$ NPLs with an $O_{h}$ cubic symmetry and a $D_{4h}$ tetragonal symmetry. Different dielectric mismatches are considered, the outside dielectric constant $\epsilon_{2}$ varying from 6.56 to 1. Figure S8: Scheme depicting how the exciton fine structure spectrum might be influenced as observed in a photoluminescence microscopy experiment (flat lying nanoplatelets).
