# Supplementary material for: Dielectric Confinement and Exciton Fine Structure in Lead Halide Perovskite Nanoplatelets
Source: Nanomaterials (Basel). 2021 Nov 13;11(11):3054. doi: 10.3390/nano11113054 (PMC8621522; doi:10.3390/nano11113054)
Supplement: Supplementary file 1 [file nanomaterials-11-03054-s001.zip › nanomaterials-1459461-supplementary(1).pdf]

Supplementary Information
Dielectric Confinement and Exciton Fine Structure in Lead Halide Perovskite Nanoplatelets

A. Ghribi,[†] R. Ben Aich,[†] K. Boujdaria,[*,†] T. Barisien,[‡] L. Legrand,[‡] M. Chamarro,[‡] C. Testelin[‡]
E-mail: kais.boujdaria@fsb.rnu.tn

November 8, 2021

[*]To whom correspondence should be addressed
[†]Université de Carthage, Faculté des Sciences de Bizerte, LR01ES15 Laboratoire de Physique des Matériaux: Structure et Propriétés, 7021 Bizerte, Tunisia.
[‡]Sorbonne Université, CNRS, Institut des NanoSciences de Paris, F-75005, Paris, France.

S1 Bulk parameters of $CsPbX_3$

We investigate the excitonic fine structure in $CsPbX_3$ NPLs with and without dielectric effects. $CsPbX_3$ ($X = Br, I, Cl$) parameters are given in Table S1. E_g is the band-gap energy, ϵ_1 is the relative dielectric constant and ϵ_X is the high-frequency dielectric constant. Some of the numerical parameters are taken from experimental studies.[1–4] In absence of data in literature, we assumed $\epsilon_X = \epsilon_\infty = 4.07$ for $CsPbCl_3$.[5] The exciton Bohr radius values, a_X, are calculated following the relationship $a_X = \frac{m_0}{\mu_X}\epsilon_1 a_0$ where $\mu_X = 0.126\ m_0$ for $CsPbBr_3$,[1] $\mu_X = 0.114\ m_0$ for $CsPbI_3$[1] and $\mu_X = 0.202\ m_0$ for $CsPbCl_3$.[2] E_{P_S} are the Kane energy for cubic phase perovskites.[6] a_0 is the Hydrogen Bohr radius.

Numerical values given in Table S2 summarize the tetragonal parameters used in this work. They are calculated using the 16-band **k.p** model in tetragonal bulk crystals.[7] We denote by Δ_C the spin-orbit coupling and T the tetragonal crystal field coupling, both defining the phase parameter, θ, according to the relationship $\tan\theta = \frac{2\sqrt{2}\Delta_C}{(\Delta_C - 3T)}$, with $0 < \theta < \frac{\pi}{2}$. $\left(E_{P_{S,\rho}}, E_{P_{S,z}}\right)$ are the related energies to the anisotropic interband momentum-matrix for the x $(= y)$ and z polarizations, $(P_{S,\rho}, P_{S,z})$. These Kane energies $\left(E_{P_{S,\rho}}, E_{P_{S,z}}\right)$ are defined as $E_{P_{S,\rho}} = \frac{2m_0}{\hbar^2}P_{S,\rho}^2$ and $E_{P_{S,z}} = \frac{2m_0}{\hbar^2}P_{S,z}^2$. m_0 denotes the free electron mass.

S2 Derivation of the Long Range Hamiltonian

Excitonic fine structure is calculated using pair states formed by the lowest electron and hole states only. The CB and VB states are respectively:

$$\left\{\begin{array}{l} \left|j_e = \frac{1}{2}, j_z^e = +\frac{1}{2}\right\rangle = i\left[-\alpha\left|X_C \downarrow\right\rangle - i\beta\left|Y_C \downarrow\right\rangle + \gamma\left|Z_C \uparrow\right\rangle\right] \\ \left|j_e = \frac{1}{2}, j_z^e = -\frac{1}{2}\right\rangle = i\left[-\alpha\left|X_C \uparrow\right\rangle + i\beta\left|Y_C \uparrow\right\rangle - \gamma\left|Z_C \downarrow\right\rangle\right] \\ \left|j_v = \frac{1}{2}, j_z^v = +\frac{1}{2}\right\rangle = \left|S_v \uparrow\right\rangle, \left|j_v = \frac{1}{2}, j_z^v = -\frac{1}{2}\right\rangle = \left|S_v \downarrow\right\rangle \end{array}\right. \tag{S1}$$

Excitonic fine structure is then calculated using pair states formes by the lowest electron and hole states only. The hole states are deduced from the VB electron states $\left|j_v = \frac{1}{2}, j_z^v = \pm\frac{1}{2}\right\rangle$ via the time-reversal operator $\mathcal{I}$. Four electron-hole pairs can be obtained (see Eq (12) of the main text). Using a basis formed by $\{\left|+1\right\rangle, \left|-1\right\rangle, \left|0_B\right\rangle; \left|0_D\right\rangle\}$, the long-range (LR) contribution to the electron-hole exchange interaction is represented by the matrix:

Table S. 1: Summary of basic physical parameters used in calculating the excitonic fine structure splittings.

Compound	E_g (eV)	ϵ_1	ϵ_X	a_X (nm)	E_{P_S} (eV)
$CsPbBr_3$[a]	2.342	7.3	4.45[c]	3.07	28.41[f]
$CsPbI_3$[a]	1.723	10	4.3[d]	4.64	23.81[f]
$CsPbCl_3$[b]	3.056	6.56	4.07[e]	1.72	29.81[f]

[a] Reference [1];[b] Reference [2];[c] Reference [3];[d] Reference [4]; [e] Reference [5];[f] Reference [6].

$$\mathcal{H}^{LR} = \begin{bmatrix} \Sigma_d & \Sigma_{od} & 0 & 0 \\ \Sigma_{od} & \Sigma_d & 0 & 0 \\ 0 & 0 & \Sigma_z & 0 \\ 0 & 0 & 0 & 0 \end{bmatrix} \tag{S2}$$

The matrix elements of the $\mathcal{H}^{LR}$ operator can be calculated according to the general expression of the LR interaction[8,9]

$$\mathcal{H}^{LR}_{\substack{m'n'\\mn}} \begin{pmatrix} \mathbf{r}'_e & \mathbf{r}'_h \\ \mathbf{r}_e & \mathbf{r}_h \end{pmatrix} = \sum_{i,j} \mathfrak{Q}^{ij}_{\substack{m'\mathcal{I}n\\ \mathcal{I}n'\ m}} \frac{\partial^2}{\partial r^i_e \partial r^j_e} \overline{\mathcal{V}}_C(\mathbf{r}_e - \mathbf{r}'_h)\ \delta\left(\mathbf{r}_e - \mathbf{r}_h\right) \delta\left(\mathbf{r}'_e - \mathbf{r}'_h\right) \tag{S3}$$

$\overline{\mathcal{V}}_C$ is easily deduced from $\mathcal{V}_C$ by replacing ϵ_1 in the denominator by ϵ_X which is the dielectric constant at the exciton resonance; m, m' (n, n') are the Bloch states of the electron in the conduction band (the hole in the valence band), and $(\mathbf{r}_e, \mathbf{r}'_e)$ and $(\mathbf{r}_h, \mathbf{r}'_h)$ denotes the coordinates of the electrons and holes, respectively. $\mathcal{I}$ is the time-reversal operator to use in the hole convention. $\mathfrak{Q}^{ij}_{\substack{m'\mathcal{I}n\\ \mathcal{I}n'\ m}}$ is given by

$$\mathfrak{Q}^{ij}_{\substack{m'\mathcal{I}n\\ \mathcal{I}n'\ m}} = \frac{\hbar^2}{m_0^2} \frac{\langle m'|\, p_i \,|\mathcal{I}n'\rangle \, \langle \mathcal{I}n|\, p_j \,|m\rangle}{\left(E^0_m - E^0_n\right)\left(E^0_{m'} - E^0_{n'}\right)}\,, \tag{S4}$$

where p_i (p_j) is the i (j) component of the $\mathbf{p}$ momentum; E^0_ν ($\nu = m, m', n, n'$) is the νth band energy.

To derive the expression of the LR Hamiltonian, it is then necessary to calculate $\left(\partial^2\overline{\mathcal{U}}_C/\partial r^i_e \partial r^j_e\right)$ and $\left(\partial^2\overline{\mathcal{W}}_C/\partial r^i_e \partial r^j_e\right)$. First, we consider the contribution of $\overline{\mathcal{U}}_C$. From the Equation (9) and in the reciprocal space (wave vector $\mathbf{q}$), one deduces:

Table S. 2: Numerical values of the tetragonal parameters used in this work. They are determined from the 16-band $\boldsymbol{k.p}$ *model [7].*

Compound	Δ_C (eV)	T (meV)	θ (°)	$E_{P_{S,z}}$ (eV)	$E_{P_{S,\rho}}$ (eV)
$CsPbBr_3$	1.49	147	40.4	15.65	16.80
$CsPbI_3$	1.43	155	39.56	17.03	19.81
$CsPbCl_3$	1.55	148	37.65	15.24	17.21

$$\frac{\partial^2 \overline{\mathcal{U}}_C}{\partial r_e^i \partial r_e^j} = \frac{1}{(2\pi)^3} \int d\mathbf{q} \left(\frac{e^2}{\epsilon_0 \epsilon_X} \frac{q_i q_j}{q^2} \right) \frac{1-\eta^4}{1+\eta^4 - 2\eta^2 \cos(2q_z L_z)} \exp i\mathbf{q}.\left(\mathbf{r}_e - \mathbf{r}_h\right) \tag{S5}$$

Recalling that $\eta = \frac{(\epsilon_1 - \epsilon_2)}{(\epsilon_1 + \epsilon_2)}$. The associate LR contribution can then be written:

$$\frac{1}{(2\pi)^3} \int d\mathbf{q} \mathbf{F}(\mathbf{q}) \mathfrak{Q}_{\substack{m'\mathcal{I}n \\ \mathcal{I}n'\ m}}(\mathbf{q}) \exp i\mathbf{q}.\left(\mathbf{r}_e - \mathbf{r}_h\right) \delta\left(\mathbf{r}_e - \mathbf{r}_h\right) \delta\left(\mathbf{r}'_e - \mathbf{r}'_h\right) \tag{S6}$$

in which one has defined the function

$$\mathbf{F}(\mathbf{q}) = \frac{e^2}{\epsilon_0 \epsilon_X} \frac{1}{q^2} \frac{1-\eta^4}{1+\eta^4 - 2\eta^2 \cos(2q_z L_z)} \tag{S7}$$

and the operator

$$\mathfrak{Q}_{\substack{m'\mathcal{I}n \\ \mathcal{I}n'\ m}}(\boldsymbol{q}) = \sum_{i,j} \mathfrak{Q}^{ij}_{\substack{m'\mathcal{I}n \\ \mathcal{I}n'\ m}}(\mathbf{q}) q_i q_j \tag{S8}$$

The explicit form of the matrix representation of $\mathfrak{Q}_{\substack{m'\mathcal{I}n \\ \mathcal{I}n'\ m}}(\mathbf{q})$ in the basis $\{|+1\rangle, |-1\rangle, |0_B\rangle; |0_D\rangle\}$ is:

$$\frac{1}{E_g^2} \begin{bmatrix} \begin{pmatrix} \alpha^2 P_{S,x}^2 q_x^2 \\ +\beta^2 P_{S,y}^2 q_y^2 \end{pmatrix} & -\begin{pmatrix} \alpha P_{S,x} q_x \\ -i\beta P_{S,y} q_y \end{pmatrix}^2 & \sqrt{2}\gamma P_{S,z} q_z \begin{pmatrix} \alpha P_{S,x} q_x \\ -i\beta P_{S,y} q_y \end{pmatrix} & 0 \\ c.c. & \begin{pmatrix} \alpha^2 P_{S,x}^2 q_x^2 \\ +\beta^2 P_{S,y}^2 q_y^2 \end{pmatrix} & -\sqrt{2}\gamma P_{S,z} q_z \begin{pmatrix} \alpha P_{S,x} q_x \\ +i\beta P_{S,y} q_y \end{pmatrix} & 0 \\ c.c. & c.c. & 2\gamma^2 P_{S,z}^2 q_z^2 & 0 \\ 0 & 0 & 0 & 0 \end{bmatrix} \tag{S9}$$

where *c.c.* denotes the complex conjugate. $(P_{S,x}, P_{S,y}, P_{S,z})$ are the nonzero matrix elements of the momentum operator $\mathbf{p}$ according to the D_{2h} point group symmetry.[10]

Using the exciton wave function and its parity, it is possible to write the contribution of $\mathcal{U}_C$:

$$\left\{ \begin{array}{l} \Sigma_d^{\mathcal{U}} = \frac{\alpha^2}{E_g^2}\frac{\hbar^2}{2m_0} E_{P_{S,x}} \int d\mathbf{q} q_x^2 \mathbf{F}(\mathbf{q}) \left| \int d\mathbf{r} \Psi(r,r) \exp i\mathbf{q}.\mathbf{r} \right|^2 \\ \quad + \frac{\beta^2}{E_g^2}\frac{\hbar^2}{2m_0} E_{P_{S,y}} \int d\mathbf{q} q_y^2 \mathbf{F}(\mathbf{q}) \left| \int d\mathbf{r} \Psi(r,r) \exp i\mathbf{q}.\mathbf{r} \right|^2 \end{array} \right. \tag{S10}$$

$$\left\{ \begin{array}{l} \Sigma_d^{\mathcal{U}} = -\frac{\alpha^2}{E_g^2}\frac{\hbar^2}{2m_0} E_{P_{S,x}} \int d\mathbf{q} q_x^2 \mathbf{F}(\mathbf{q}) \left| \int d\mathbf{r} \Psi(r,r) \exp i\mathbf{q}.\mathbf{r} \right|^2 \\ \quad + \frac{\beta^2}{E_g^2}\frac{\hbar^2}{2m_0} E_{P_{S,y}} \int d\mathbf{q} q_y^2 \mathbf{F}(\mathbf{q}) \left| \int d\mathbf{r} \Psi(r,r) \exp i\mathbf{q}.\mathbf{r} \right|^2 \end{array} \right. \tag{S11}$$

$$\left\{ \Sigma_z^{\mathcal{U}} = \frac{2\gamma^2}{E_g^2}\frac{\hbar^2}{2m_0} E_{P_{S,z}} \int d\mathbf{q} q_z^2 \mathbf{F}(\mathbf{q}) \left| \int d\mathbf{r} \Psi(r,r) \exp i\mathbf{q}.\mathbf{r} \right|^2 \right. \tag{S12}$$

where $E_{P_{S,j}} = (2m_0/\hbar^2)\, P_{S,j}^2$ $(j = x, y, z)$.

The contributions $\Sigma_\lambda^{\mathcal{W}}$ $(\lambda = o, od, z)$ of $\overline{\mathcal{W}}_C$ will depend on:

$$\left\{ \begin{array}{l} \frac{\partial^2 \overline{\mathcal{W}}_C}{\partial r_e^i \partial r_e^j} = \frac{1}{(2\pi)^3} \int d\mathbf{q} \left(\frac{e^2}{\epsilon_0 \epsilon_X} \frac{q_i q_j}{q^2} \right) \frac{2\eta(1-\eta^2)\cos(q_z L_z)}{1+\eta^4 - 2\eta^2 \cos(2 q_z L_z)} \\ \qquad \times \exp\left[i\mathbf{q}_\parallel . (\boldsymbol{\rho}_e - \boldsymbol{\rho}_h) + i q_z (z_e + z_h) \right] \end{array} \right. \tag{S13}$$

Doing a similar derivation, the associate LR contribution can then be written:

$$\frac{1}{(2\pi)^3} \int d\mathbf{q} \mathbf{G}(\mathbf{q}) \mathfrak{Q}_{\substack{m' \mathcal{I} n \\ \mathcal{I} n' \, m}}(\mathbf{q}) \exp\left[i\mathbf{q}_\parallel . (\boldsymbol{\rho}_e - \boldsymbol{\rho}_h) + i q_z (z_e + z_h) \right] \delta\left(\mathbf{r}_e - \mathbf{r}_h \right) \delta\left(\mathbf{r}'_e - \mathbf{r}'_h \right) \tag{S14}$$

where one has defined the function

$$\mathbf{G}(\mathbf{q}) = \frac{e^2}{\epsilon_0 \epsilon_X} \frac{1}{q^2} \frac{2\eta(1-\eta^2)\cos(q_z L_z)}{1+\eta^4 - 2\eta^2 \cos(2 q_z L_z)} \tag{S15}$$

The contributions of $\overline{\mathcal{W}}_C$, namely $\Sigma_\lambda^{\mathcal{W}}$ can then be derived and are similar to $\Sigma_\lambda^{\mathcal{U}}$, with the square $\mathbf{r}$-integral $\left| \int d\mathbf{r} \Psi(r,r) \exp i\mathbf{q}.\mathbf{r} \right|^2$ replaced by

$$\int d\mathbf{r}\Psi(r,r)\exp i\mathbf{q}.\mathbf{r}\int d\mathbf{r}\Psi(r,r)\exp i\mathbf{q}.\mathfrak{r} \tag{S16}$$

with $\mathbf{r}=(x,y,z)$ and $\mathfrak{r}=(x,y,-z)$. Due to the parity of the function $\Psi(\mathbf{r},\mathbf{r})$ both integrales are equal. This leads to the same expressions as for $\Sigma_\lambda^{\mathcal{U}}$, with $\mathbf{F}(\mathbf{q})$ replaced by $\mathbf{G}(\mathbf{q})$.

Finally, the LR coupling coefficients Σ_λ are written according to eqs (S9-S11) with $\mathbf{F}(\mathbf{q})$ replaced by

$$\mathcal{D}(\mathbf{q})=\frac{e^2}{\epsilon_0\epsilon_X}\frac{1}{q^2}\frac{(1-\eta^2)\cos(q_zL_z)}{1+\eta^2-2\eta\cos(q_zL_z)} \tag{S17}$$

and it is then possible to estimate the whole LR couplings:

$$\begin{cases} \Sigma_d=\left(\alpha^2E_{P_{S,x}}I_x+\beta^2E_{P_{S,,y}}I_y\right)\Lambda\frac{3\pi^2a_X^3|\mathcal{N}(a,\zeta)|^2}{L_z} \\ \Sigma_{od}=\left(-\alpha^2E_{P_{S,x}}I_x+\beta^2E_{P_{S,,y}}I_y\right)\Lambda\frac{3\pi^2a_X^3|\mathcal{N}(a,\zeta)|^2}{L_z} \\ \Sigma_z=2\gamma^2E_{P_{S,z}}I_z\Lambda\ \frac{3\pi^2a_X^3|\mathcal{N}(a,\zeta)|^2}{L_z} \end{cases} \tag{S18}$$

with $\Lambda=\frac{1}{3E_g^2}\frac{\hbar^2}{2m_0}\frac{e^2}{\epsilon_0\epsilon_X}\frac{1}{\pi a_X^3}$. The integrals I_j $(j=x,y,z)$ are written as

$$I_x=\int d\mathbf{u}\frac{r^2s^2u_x^2}{r^2s^2u_x^2+s^2u_y^2+r^2u_z^2}\frac{\sin^2u_x}{u_x^2}\frac{\sin^2u_y}{u_y^2}\frac{\sin^2u_z}{\left(u_z^2-\frac{\pi^2}{4}\right)^2}\mathfrak{d}(u_z) \tag{S19}$$

$$I_y=\int d\mathbf{u}\frac{s^2u_y^2}{r^2s^2u_x^2+s^2u_y^2+r^2u_z^2}\frac{\sin^2u_x}{u_x^2}\frac{\sin^2u_y}{u_y^2}\frac{\sin^2u_z}{\left(u_z^2-\frac{\pi^2}{4}\right)^2}\mathfrak{d}(u_z) \tag{S20}$$

$$I_z=\int d\mathbf{u}\frac{r^2u_z^2}{r^2s^2u_x^2+s^2u_y^2+r^2u_z^2}\frac{\sin^2u_x}{u_x^2}\frac{\sin^2u_y}{u_y^2}\frac{\sin^2u_z}{\left(u_z^2-\frac{\pi^2}{4}\right)^2}\mathfrak{d}(u_z) \tag{S21}$$

in which $\mathfrak{d}(u_z)=\frac{(1-\eta^2)}{1+\eta^2-2\eta\cos(2u_z)}$. $r=L_y/L_x$ and $s=L_z/L_x$ are the anisotropy parameters.

S3 Coulomb interaction enhancement factor and self-energy potential in $CsPbBr_3$ NPLs

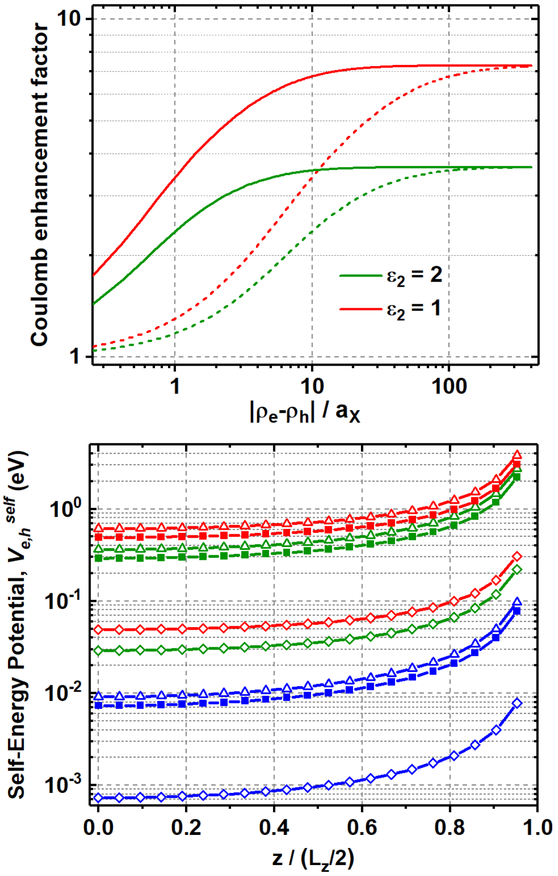

Figure S. 1: Upper panel: Enhancement factor as the ratio of the modified Coulomb potential (Eq. (7) in main text) to the 'usual' e-h electrostatic potential, $\mathcal{V}_C(\mathbf{r}_e, \mathbf{r}_h) = -\frac{e^2}{4\pi\epsilon_0\epsilon_1}\frac{1}{|\mathbf{r}_e - \mathbf{r}_h|}$, *for* $z_e = z_h = 0$ *(median NPL plane) in* $CsPbBr_3$ *(*$\epsilon_1 = 7.3$*); solid lines:* $L_z = 2.90$ *nm* $\approx a_X$ *(5 ML); dash lines:* $L_z \approx 10 \times a_X$*. As expected, the enhancement factor reaches* $\frac{\epsilon_1}{\epsilon_2}$ *at long inter-particles distances with a more extended transition for larger thicknesses NPLs. Lower panel: plots of the 'self-energy' potential (Eq. (6) in main text) in a* $CsPbBr_3$ *NPL as a function of the NPL thickness (*$\triangle$*= 4 ML,* $\blacksquare$ *= 5 ML and* $\diamond$ *= 50 ML) and outer dielectric constant,* ϵ_2 *(red curve:* $\epsilon_2 = 1$*, green curve:* $\epsilon_2 = 2$ *and blue curve:* $\epsilon_2 = 7.0$*).*

S4 Near band-gap energies of $CsPbI_3$ and $CsPbCl_3$ NPLs

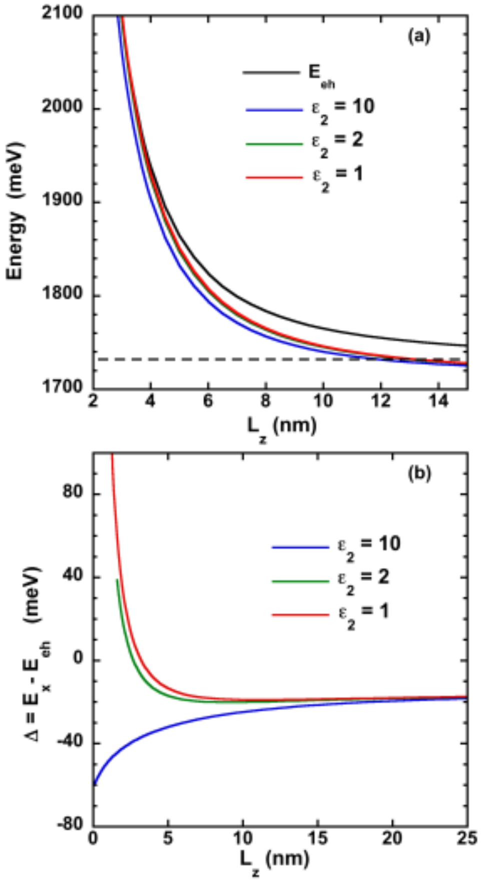

Figure S. 2: (a) Free e-h pair energy E_{eh} (black line) and excitonic energy E_X with different dielectric mismatches, for $CsPbI_3$. The dashed line correspond to the bulk gap energy E_g. (b) Energy difference $\Delta = E_X - E_{eh}$ with different dielectric mismatches.

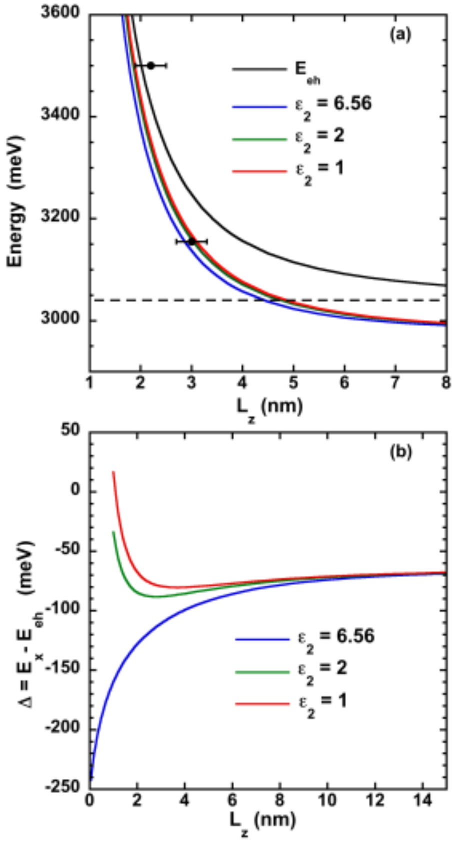

Figure S. 3: (a) Free e-h pair energy E_{eh} (black line) and excitonic energy E_X with different dielectric mismatches, for $CsPbCl_3$. The dashed line correspond to the bulk gap energy E_g. The black symbols are experimental data from Ref.[11] and Ref.[12] (b) Energy difference $\Delta = E_X - E_{eh}$ with different dielectric mismatches.

S5 Exciton binding energy of $CsPbI_3$ and $CsPbCl_3$ NPLs

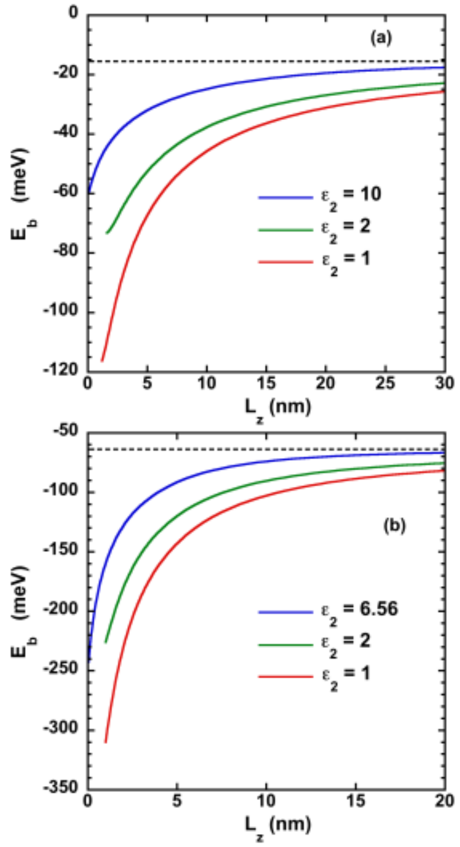

Figure S. 4: Binding energy E_b of the e-h pair including the Coulomb interaction and the dielectric effects with different dielectric mismatches, in (a) $CsPbI_3$ NPLs and (b) $CsPbCl_3$ NPLs. The dashed line corresponds to the bulk binding energy R: (a) R = -15.5 meV; (b) R = -63.4 meV.

S6 Wave function normalization factor and variational Bohr Radius versus the NPL thikness for $CsPbBr_3$

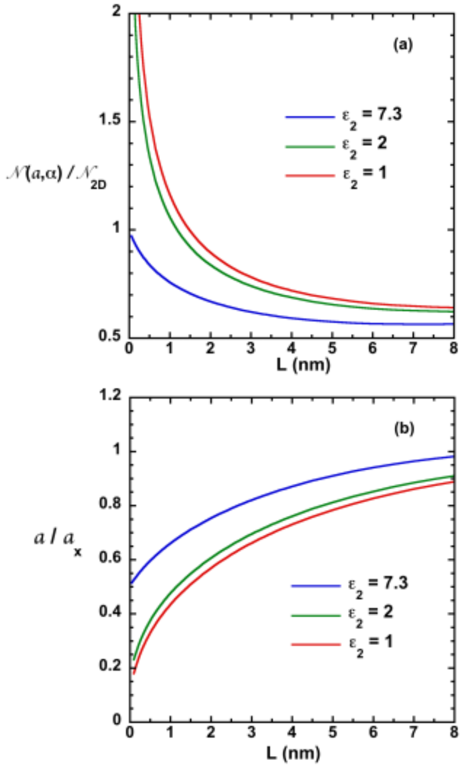

Figure S. 5: (a) Normalization factor $\mathcal{N}(a,\zeta)$ of the trial function versus the thickness L_z with different dielectric mismatches, for $CsPbBr_3$ NPL. $\mathcal{N}(a,\zeta)$ is normalized by the 2D factor $\mathcal{N}_{2D} = \sqrt{\frac{8}{\pi a_X^2}}$ associated to the exciton wave function in a perfect 2D system. (b) Effective Bohr radius normalized by the bulk Bohr radius a_X.

In absence of dielectric effect, the wave function normalization factor $\mathcal{N}(a,\zeta)$ tends to $\mathcal{N}_{2D} = \sqrt{\frac{8}{\pi a_X^2}}$, the value associated to the exciton wave function in a perfect 2D system ($L_z = 0$), as confirmed by the blue curve

($\varepsilon_2 = 7.3$) on Figure S.5a. The effective Bohr radius, a, shows the expected behavior, converging to $\frac{a_X}{2}$ for $L_z = 0$ (see Figure S.5b). In presence of dielectric effects, the coefficient $\mathcal{N}(a,\zeta)$ increases with $\eta = \frac{\varepsilon_1-\varepsilon_2}{\varepsilon_1+\varepsilon_2}$, giving an extra contribution to the fine structure splitting. Due to the single self energy potential induced by the image charges, a lateral confinement appears, reducing the effective Bohr radius as shown on Figure S.5b.

S7 Fine structure splitting for $CsPbI_3$ NPLs in the cubic and tetragonal symmetry

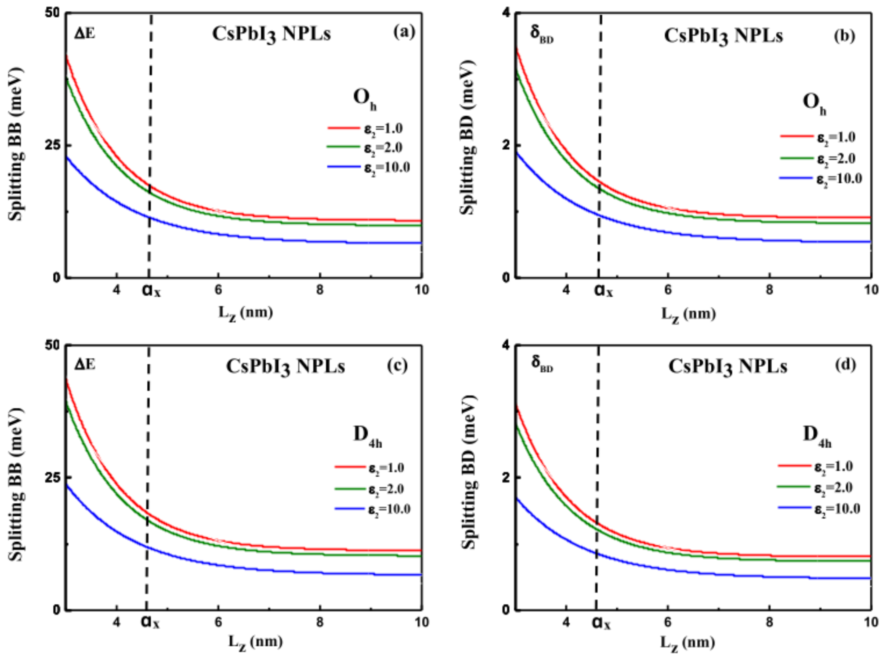

Figure S. 6: Bright-Bright splitting ΔE and Bright-Dark splitting δ_{BD}, in $CsPbI_3$ NPLs with an O_h cubic symmetry (upper curves) and a D_{4h} tetragonal symmetry (lower curves). Different dielectric mismatches are considered, the outside dielectric constant ε_2 varying from 10 to 1. The vertical line gives the value of the exciton Bohr radius.

S8 Fine structure splitting for $CsPbCl_3$ in the cubic and tetragonal symmetry

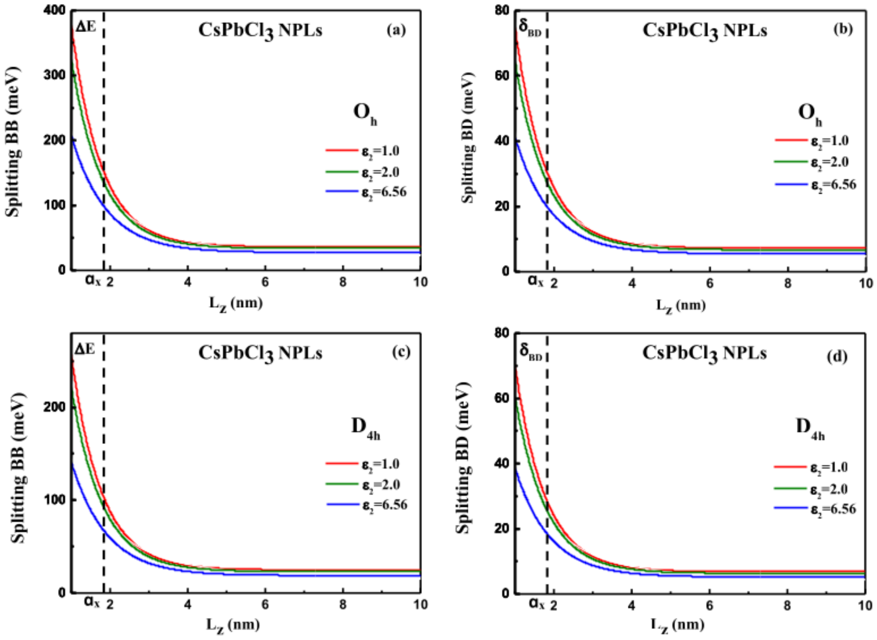

Figure S. 7: Bright-Bright splitting ΔE and Bright-Dark splitting δ_{BD}, in $CsPbCl_3$ NPLs with an O_h cubic symmetry (upper curves) and a D_{4h} tetragonal symmetry (lower curves). Different dielectric mismatches are considered, the outside dielectric constant ε_2 varying from 6.56 to 1. The vertical line gives the value of the exciton Bohr radius.

S9 Energy levels configuration: a summary

To be comprehensive, four cases should be considered. For each symmetry, D_{4h} (tetragonal) or D_{2h} (orthorhombic), the fine structure spectrum will depend on the $\boldsymbol{c}$ axis orientation: **(i)** orthogonal to the platelet plane (Z orthogonal to the platelet plane if using the notation of the main text), or **(ii)** $\boldsymbol{c}$ lying in the platelet plane.

The energy levels are obtained by the diagonalization of the SR and LR interaction Hamiltonian given by equations (14) and (15). The resulting excitonic structures are briefly described below.

Please note that the eigenenergies calculated below (Σ_+, Σ_-, Σ_k with $k = X, Y, Z$) are absolute energies that place the fine structure levels on an absolute scale with a ground dark state setting the zero (energy $\Sigma_0 = 0$). In contrast ΔE, ΔE_1 and ΔE_2 used in the main text are energy differences. For clarity reasons, and as a reminder, their definition are provided in each of the addressed cases.

We finally emphasize that, with the used convention for the $|X\rangle$ and $|Y\rangle$ states (i.e. $|X\rangle = \frac{1}{\sqrt{2}}[|{+1}\rangle - |{-1}\rangle]$, $|Y\rangle = \frac{1}{\sqrt{2}}[|{+1}\rangle + |{-1}\rangle]$), the dipoles associated to $|X\rangle$, $|Y\rangle$ and $|Z\rangle$ oscillate along the x, y and z physical axis, respectively.

1. D_{4h}, Z ($= c$ axis) orthogonal to the NPL plane.

   In the 2D limit, $I_x = I_y = 0$ and $I_z \neq 0$ in the Σ_d and Σ_{od} terms (LR interaction matrix). We thus obtain:

$$\Sigma_d = \Sigma_{od} = 0 \text{ and } \Sigma_Z = 2\gamma^2 E_{P_{S,z}} I_z \Lambda \frac{3\pi^2 a_X^3}{Lz} |\mathcal{N}(a,\zeta)|^2 \qquad \text{(S22)}$$

   Moreover $\alpha^2 = \beta^2 = \frac{\cos^2\theta}{2}$ and $\gamma^2 = \sin^2\theta$ (see main text).
   Summing the LR and SR contributions, one finds *for the eigenvalues* (equation (20) in the main text):

$$\begin{cases} \Sigma_\pm = \frac{9}{4}\Delta_{SR}\pi a_X^3 \frac{|\mathcal{N}(a,\zeta)|^2}{L_z}\cos^2\theta & \text{for } |\pm 1\rangle \\ \Sigma_Z = \left[\frac{9}{4}\Delta_{SR} + 3E_{P_{S,z}} I_z \pi\Lambda\right]\pi a_X^3 \frac{|\mathcal{N}(a,\zeta)|^2}{L_z} \times 2\sin^2\theta & \text{for } |0_B\rangle \end{cases} \qquad \text{(S23)}$$

   The fine structure consists of a degenerate doublet, $|\pm 1\rangle$ and a higher energy singlet, $|Z\rangle$. We find that $\Sigma_+ = \Sigma_- \ll \Sigma_Z$ and ΔE=$\Sigma_Z - \Sigma_\pm$. Here (and below) '$\ll$' means that the energy of the $|0_B\rangle$ state is significantly larger.

2. D_{4h}, Z parallel to the NPL plane.

   One has to consider $I_x = I_z = 0$ and $I_y \neq 0$. y (and the dipole

associated to the $|Y\rangle$ state) is orthogonal to the NPL plane. This leads to:

$$\Sigma_d = \Sigma_{od} = \beta^2 E_{P_{S,y}} I_y \Lambda \frac{3\pi^2 a_X^3}{L_z} |\mathcal{N}(a,\zeta)|^2 \text{ and } \Sigma_Z = 0 \tag{S24}$$

Taking into account the SR interaction contribution (and noticing that α, β and γ parameters, that depend on the symmetry, are unchanged as compared to the previous case), one finds the eigenenergies:

$$\begin{cases} \Sigma_Y = \left[\frac{9}{4}\Delta_{SR} + 3E_{P_{S,y}} I_y \pi \Lambda\right] \frac{\pi a_X^3}{L_z} |\mathcal{N}(a,\zeta)|^2 \cos^2\theta & \text{for } |Y\rangle \\ \Sigma_X = \frac{9}{4}\Delta_{SR} \frac{\pi a_X^3}{L_z} |\mathcal{N}(a,\zeta)|^2 \cos^2\theta & \text{for } |X\rangle \\ \Sigma_Z = \frac{9}{4}\Delta_{SR} \frac{\pi a_X^3}{L_z} |\mathcal{N}(a,\zeta)|^2 \times 2\sin^2\theta & \text{for } |Z\rangle \end{cases} \tag{S25}$$

$\Sigma_X \neq \Sigma_Z$ and the states degeneracy is fully lifted. The state having its dipole orthogonal to the NPL plane is the highest energy one.
We note that $\Sigma_X < \Sigma_Z \ll \Sigma_Y$. Here the smaller splitting will be noted $\Delta E_1 = E_Z - E_X$ while the larger is $\Delta E_2 = E_Y - E_Z$. We refer the reader to the main text and to the first sections of the SI for a quantitative estimation of the energy differences in $CsPbBr_3$ and $CsPbCl_3$, $CsPbI_3$ compounds respectively.

3. D_{2h}, Z orthogonal to the NPL plane.

   First we take I_x=I_y=0 and $I_z \neq$0 to evaluate the Σ_d and Σ_{od} terms (LR interaction contribution). They are given by eq. (S22) . Then one has to diagonalize the SR interaction hamiltonian in its 'more general form' with $\alpha \neq \beta \neq \gamma$. A basic derivation provides the eigenvalues $2\alpha^2$, $2\beta^2$ and $2\gamma^2$ for the matrix part in (14). The final eigenenergies thus reads (equation (22) in the main text):

$$\begin{cases} \Sigma_Y = \frac{9}{2}\Delta_{SR} \pi a_X^3 \frac{|\mathcal{N}(a,\zeta)|^2}{L_z} \beta^2 & \text{for } |Y\rangle = \frac{1}{\sqrt{2}}\left[|{+1}\rangle + |{-1}\rangle\right] \\ \Sigma_X = \frac{9}{2}\Delta_{SR} \pi a_X^3 \frac{|\mathcal{N}(a,\zeta)|^2}{L_z} \alpha^2 & \text{for } |X\rangle = \frac{1}{\sqrt{2}}\left[|{+1}\rangle - |{-1}\rangle\right] \\ \Sigma_Z = \left[\frac{9}{2}\Delta_{SR} + 6E_{P_{S,z}} I_z \pi \Lambda\right] \pi a_X^3 \frac{|\mathcal{N}(a,\zeta)|^2}{L_z} \gamma^2 & \text{for } |Z\rangle \end{cases} \tag{S26}$$

   Thus a triplet is also predicted with $\Sigma_X < \Sigma_Y \ll \Sigma_Z$. The splittings in energy are $\Delta E_1 = E_Y - E_X$ and $\Delta E_2 = E_Z - E_Y$.

4. D_{2h}, Z parallel to the NPL plane.

y chosen orthogonal to the NPL plane implies that I_x=I_z=0 and I_y ≠0. Σ_d, Σ_{od} and Σ_Z are already known (see eq. (S24)). When incorporating the SR interaction contribution, one gets, after diagonalization:

$$\begin{cases} \Sigma_Y = \left[\frac{9}{4}\Delta_{SR} + 3E_{P_{S,y}} I_y \pi\Lambda\right] \frac{\pi a_X^3}{L_z} |\mathcal{N}(a,\zeta)|^2 2\beta^2 & \text{for } |Y\rangle \\ \Sigma_X = \frac{9}{4}\Delta_{SR}\frac{\pi a_X^3}{L_z}|\mathcal{N}(a,\zeta)|^2 2\alpha^2 & \text{for } |X\rangle \\ \Sigma_Z = \frac{9}{4}\Delta_{SR}\frac{\pi a_X^3}{L_z}|\mathcal{N}(a,\zeta)|^2 2\gamma^2 & \text{for } |Z\rangle \end{cases} \tag{S27}$$

In this case, $\Sigma_X \neq \Sigma_Z$ and, as $\alpha^2 < \gamma^2$, $\Sigma_X < \Sigma_Z \ll \Sigma_Y$. Again the degeneracy is lifted and the bright exciton fine structure consists of a triplet (splittings $\Delta E_1 = E_Z - E_X$ and $\Delta E_2 = E_Y - E_Z$).

S10 Estimation of the depolarisation factors in 2D geometry

To predict the NPL optical (experimental) response in emission one needs to take into account the manner the applied field might be reduced inside the nanostructure due to the strong dielectric mismatch that exists between the inside and the outside. Such effects ('local field effetcs') are taken into account in the flattened ellipsoid model that provides an access to analytical formula when estimating the attenuation factor of the electromagnetic field (for example the approach was successfully used in ref[13] and ref[14]). The main elements of the theory can be found in the work of A. Rodina and A. Efros.[15]

When an emitting dipole is set along the direction $\mu = x$, y or z (z is perpendicular to the NPL plane), the probability to emit light is reduced by the screening factor $\mathcal{D}_\mu$:

$$\mathcal{D}_\mu = \left[\frac{1}{1 + n^\mu(k-1)}\right]^2, \tag{S28}$$

where n^μ is the depolarization factor for the μ direction, and k is the ratio between the relative dielectric constants - inside and outside the NPL - taken at the emission wavelength: $k = \epsilon^{in}/\epsilon^{out}(\lambda_{em})$.

In the 2D limit, $n^x = n^y = 0$ and $n^z = 1$ so that $\mathcal{D}_x = \mathcal{D}_y = 1$ and $\mathcal{D}_z = 1/k^2 = [\epsilon^{out}/\epsilon^{in}]^2$.

Considering $\epsilon^{in} \sim 7$ for $CsPbBr_3$ as determined in,[16] $\mathcal{D}_z$ ranges from $\sim 1/50$ to $1/12$, as ϵ^{out} varies from 1 to 2. The coupling of the light with a z oriented dipole is then weakened within a factor that is at least $\sim$10 as compared to the in-plane coupling. Let us note that the calculations might be carried out in the situation of large (non infinite along x and y) platelets as in ref[13] and ref[14] showing that the results are very poorly affected as long as $L_{x,y} \gg L_z$.

S11 How the NPL exciton fine structure spectrum might show up in a micro-PL experiment

Three main effects will play a crucial role in 'shaping' the optical response of the NPLs in a micro-photoluminescence experiment. They are presented here as operating sequentially: **(i)** fast energy transfer (E.T.) inside the fine structure manifold that will quench the higher energy state emission at low temperature; **(ii)** the local field effect that will have a drastic impact, reducing the internal field inside the nanostructure i.e. the light-dipole coupling. The effect will be significant along the highly confined direction (z or y in the Figure S.8); **(iii)** the detection geometry: due to the dipolar emission pattern, a poor photon collection will be achieved in the direction aligned with the emitting dipole axis. The polar diagram - right side of Figure S.8 - provides the angle distribution of the radiated intensity that would be measured on a photodetector placed behind an analyzer in the detection path (along the optical axis). (a) and (b) are for D_{4h} symmetry while (c) and (d) relate to the D_{2h} symmetry. In (b) and (d) the c cristallographic axis lies in the platelet while it is perpendicular to it in the configurations (a) and (c).

The possible optical signatures consist either of a single line (with an isotropic distribution of the intensity in the plane perpendicular to the optical axis, either of a doublet with linear crossed polarizations). The doublet might be observed if the homogeneous broadening allows it. The expected splitting, ΔE_1, is in the meV range for platelets having a thickness of few MLs (see main text). Using the previous results, it is also seen that 'edge-lying' NPLs would always contribute in the form of a single linearly polarized line.

Note that the correct scaling in the PL peak intensity is not the one used in the Figure S.8. A 10^2 to 10^3 attenuation factor can easily result from the

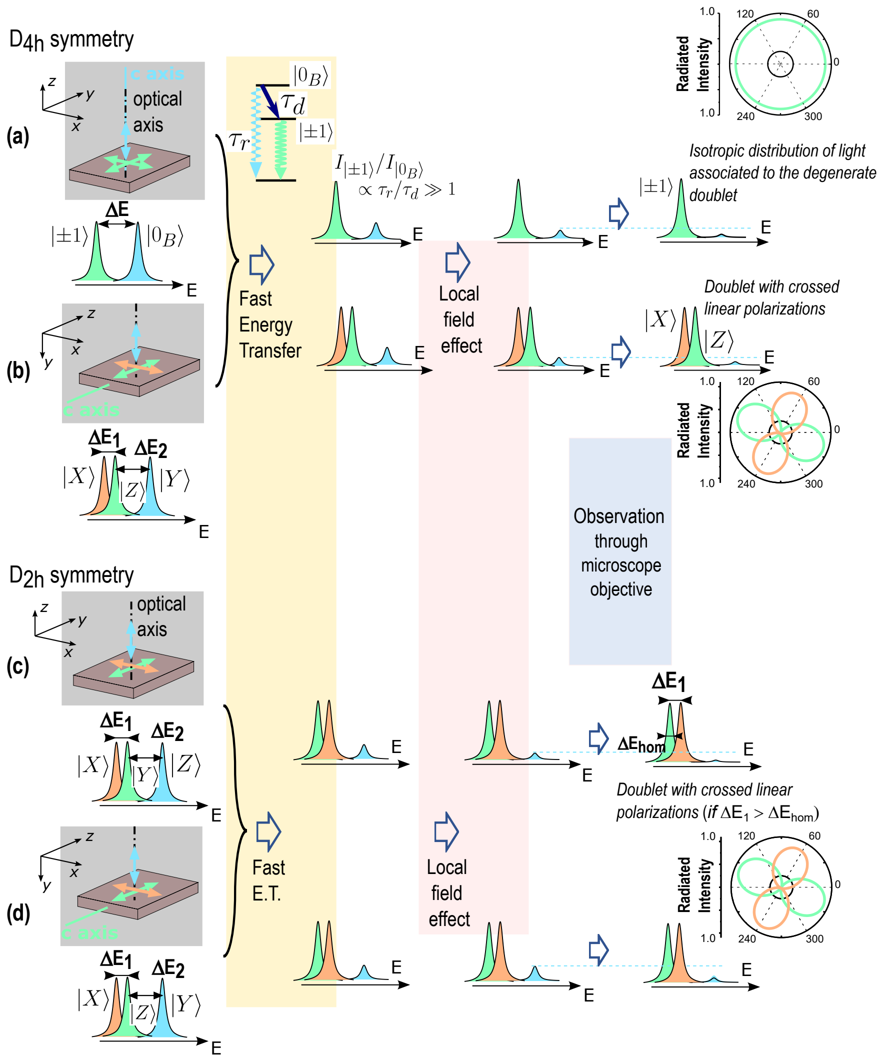

Figure S. 8: Scheme depicting how the exciton fine structure spectrum might be influenced as observed in a photoluminescence microscopy experiment (flat lying nanoplatelets).

E.T. process at low temperature (τ_d in the ps/sub ps range and τ_r in the hundred of ps/ns range). In the quantum well limit (infinite extension along x and y), the depolarization inside the structure leads to an apparent ~12 times weakening of the emitting dipole aligned along the direction of strong confinement[15] (considering the typical values $\varepsilon_{in} \sim 7.0$ and $\varepsilon_{out} \sim 2.0$.[16]).

References

(1) Yang, Z.; Surrente, A.; Galkowski, K.; Miyata, A.; Portugall, O.; Sutton, R. J.; Haghighirad, A. A.; Snaith, H. J.; Maude, D. K.; Plochocka, P.; et al. Impact of the Halide Cage on the Electronic Properties of Fully Inorganic Cesium Lead Halide Perovskite. *ACS Energy Lett.* **2017**, 2, 1621-1627.

(2) Baranowski, M.; Plochocka, P.; Sui, R.; Legrand, L.; Barisien, T.; Bernardot, F.; Xiong, Q.; Testelin, C.; Chamarro, M. Exciton Binding Energy and Effective Mass of $CsPbCl_3$: a Magneto-Optical Study. *Photonics Research* **2020**, 8, A50-A55.

(3) Chen, X.; Wang, Y.; Song, J., Li, X.; Xu, J.; Zeng, H.; Sun, H. Temperature Dependent Reflectance and Ellipsometry Studies on a $CsPbBr_3$ Single Crystal. *J. Phys. Chem. C* **2019**, 123, 16, 10564–10570.

(4) Singh, R. K.; Kumar, R.; Jain, N.; Dash, S. R.; Singh, J.; Srivastava, A. Investigation of Optical and Dielectric Properties of $CsPbI_3$ Inorganic Lead Iodide Perovskite thin Film. *Journal of the Taiwan Institute of Chemical Engineers* **2019**, 96, 538–542.

(5) Protesescu, L.; Yakunin, S.; Bodnarchuk, M. I.; Krieg, F.; Caputo, R.; Hendon, C. H.; Yang, R. X.; Walsh, A.; Kovalenko, M. V. Nanocrystals of Cesium Lead Halide Perovskites ($CsPbX_3$, X = Cl, Br, and I): Novel Optoelectronic Materials Showing Bright Emission with Wide Color Gamut. *Nano Lett.* **2015**, 15, 3692-3696.

(6) Steinmetz, V.; Ramade, J.; Legrand, L.; Barisien, T.; Bernardot, F.; Lhuillier, E.; Bernard, M.; Vabre, M.; Saïdi, I.; Ghribi, A.; et al. Anisotropic Shape of $CsPbBr_3$ Colloidal Nano-Crystals: from 1D to 2D Confinement Effects. *Nanoscale* **2020**, 12, 18978-18986.

(7) Ben Aich, R.; Ben Radhia, S.; Boujdaria, K.; Chamarro, M.; Testelin C. Multiband **k.p** Model for Tetragonal Crystals: Application to Hybrid Halide Perovskite Nanocrystals. *J. Phys. Chem. Lett.* **2020**, 11, 808-817.

(8) Tong, H.; Wu, M. W. Theory of Excitons in Cubic III-V Semiconductor GaAs, InAs and GaN Quantum Dots: Fine structure and Spin Relaxation. *Phys. Rev. B: Condens. Matter Mater. Phys.* **2011**, *83*, 235323.

(9) Gupalov, S. V. ; Ivchenko, E. L. The Fine Structure of Excitonic Levels in CdSe Nanocrystals. *Phys. Solid State* **2000**, 42, 2030-2038.

(10) Ben Aich, R.; Saïdi, I.; Ben Radhia, S.; Boujdaria, K.; Barisien, T.; Legrand, L.; Bernardot, F.; Chamarro, M.; Testelin, C. Bright-Exciton Splittings in Inorganic Cesium Lead Halide Perovskite Nanocrystals. *Phys. Rev. Appl.* **2019**, 11, 034042.

(11) Mir, W. J. ; Jagadeeswararao, M.; Das, S.; Nag, A. Colloidal Mn-Doped Cesium Lead Halide Perovskite Nanoplatelets. *ACS Energy Lett.* **2017**, 2, 537-543.

(12) Li, Z.-J.; Hofman, E.; Davis, A. H.; Khammang, A.; Wright, J. T.; Dzikovski, B.; Meulenberg, R. W.; Zheng, W. Complete Dopant Substitution by Spinodal Decomposition in Mn-Doped Two-Dimensional $CsPbCl_3$ Nanoplatelets. *Chem. Mater.* **2018**, 30, 6400-6409.

(13) Pandya, R.; Steinmetz, V.; Puttisong, Y.; Dufour, M.; Chen, W. M.; Chen, R. Y. S.; Barisien, T.; Sharma, A.; Lakhwani, G.; Mitioglu, A.; Christianen, P. C. M.; Legrand, L.; Bernardot, F.; Testelin, C.; Chin, A. W.; Ithurria, S.; Chamarro, M.; Rao, A. Fine Structure and Spin Dynamics of Linearly Polarized Indirect Excitons in Two-Dimensional CdSe/CdTe Colloidal Heterostructures. *ACS Nano* **2019**, 13, 10140-10153.

(14) Geiregat, P.; Rodá, C.; Tanghe, I.; Singh, S.; Di Giacomo, A.; Lebrun, D.; Grimaldi, G.; Maes, J.; Van Thourhout, D.; Moreels, I.; Houtepen, A. J.; Hens, Z. Localization-Limited Exciton Oscillator Strength in Colloidal CdSe Nanoplatelets Revealed by the Optically Induced Stark Effect. *Light Sci. Appl.* **2021**, 10, 112-122.

(15) Rodina, A. V.; Efros, A. L. Effect of Dielectric Confinement on Optical Properties of Colloidal Nanostructures. *J. Exp. Theor. Phys.* **2016**, 122, 554-566.

(16) Yang, Z.; Surrente, A.; Galkowski, K.; Miyata, A.; Portugall, O.; Sutton, R. J.; Haghighirad, A. A.; Snaith, H. J.; Maude, D. K.; Plochocka,

P.; Nicholas R. J. Impact of the Halide Cage on the Electronic Properties of Fully Inorganic Cesium Lead Halide Perovskites. *ACS Energy Lett.* **2017**, 2, 1621-1627.
